# Characterisation of a phylogenetically distinct PL25 family ulvan lyase from a seaweed biomass enriched metagenome

**DOI:** 10.1111/febs.70390

**Published:** 2026-01-21

**Authors:** Andrius Jasilionis, Pavithra Sivakumar, Justyna M. Dobruchowska, Sune Fjermedal, Hörður Guðmundsson, Bjorn Thor Adalsteinsson, Guðmundur Ó. Hreggviðsson, Anne S. Meyer, Eva Nordberg Karlsson

**Affiliations:** ^1^ Division of Biotechnology and Applied Microbiology, Department of Process and Life Science Engineering Lund University Sweden; ^2^ Department of Chemical Biology and Drug Discovery Utrecht Institute for Pharmaceutical Sciences, Bijvoet Center for Biomolecular Research, Utrecht University The Netherlands; ^3^ Department of Biotechnology and Biomedicine Technical University of Denmark Kongens Lyngby Denmark; ^4^ Biotechnology Group, Matís ohf Reykjavik Iceland; ^5^ Faculty of Life and Environmental Sciences University of Iceland Reykjavik Iceland

**Keywords:** enriched metagenome, PL25 family, ulvan enzymatic depolymerisation, ulvan lyase, ulvan oligosaccharides

## Abstract

Ulvan is a polysaccharide most abundant in green macroalgae biomass. Investigation of ulvan confirmed the potential of the polysaccharide for food, pharmaceutical and chemistry applications, emphasising the beneficial properties of ulvan oligosaccharides. Efficient production of oligosaccharides requires action of ulvan lyases capable of ensuring polysaccharide enzymatic depolymerisation. The armoury of available ulvan lyases was expanded by characterisation of SH2L_Ulv3 ulvan lyase, which was found to be phylogenetically distinct from previously characterised lyases attributed to PL25 family. A gene encoding a novel ulvan lyase was identified among sequences from a seaweed biomass metagenome enriched in an intertidal coastal hot spring. Identified ulvan lyase was most similar to a hypothetical protein from a Bacteroidales bacterium. Recombinant SH2L_Ulv3 was heterologously (over)produced in *Escherichia coli* at a high yield, remaining soluble in the expression host as well as after affinity purification. Ulvan lyase active as a 48.6 kDa monomer with evaluated activity optimum pH 7.5 and 200 mm NaCl at 25 °C demonstrated broad substrate specificity. SH2L_Ulv3 degraded ulvan from blade‐thallus as well as tubular‐thallus morphology algae species, efficiently producing three different DP4 and DP2 unsaturated oligosaccharides. The kinetic parameters of SH2L_Ulv3 were *K*_M_ 3.63 ± 0.12 mg·mL^−1^, *V*_max_ 1.78 ± 0.04 μmol·min^−1^·mL^−1^ and *k*_cat_ 1.46 ± 0.04 s^−1^. Magnesium ion stimulated SH2L_Ulv3 activity. The characterised enzyme was not thermostable, displaying *T*_m_ 42 °C. The computationally modelled structure of SH2L_Ulv3 revealed structural organisation and active site architecture as well as ligand substrate binding and zinc ion coordinating residues typical for PL25 lyases; however, with a larger central active site cleft facilitating ulvan polysaccharide degradation.

## Graphical abstract

Ulvan is a polysaccharide available from green seaweed with beneficial properties for various applications. The full potential of ulvan requires enzymatic degradation of the polymer, thus producing ulvan oligosaccharides. This study expands the armoury of characterised ulvan lyases. The characterised PL25 ulvan lyase, identified from a seaweed biomass metagenome enriched in an intertidal coastal hot spring, has distinct features that ensure efficient ulvan enzymatic treatment into tetrasaccharides and disaccharides.

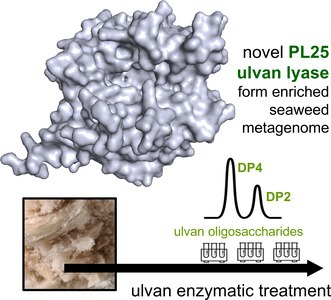

**Abbreviations**
DNS: dinitrosalicylic acid
DP2: ulvan disaccharides
DP4: ulvan tetrasaccharides
HMBC: heteronuclear multiple bond correlation
HSQC: heteronuclear single quantum coherence
ICP‐OES: inductively coupled plasma optical emission spectroscopy
IPTG: isopropyl β‐d‐1‐thiogalactopyranoside
nanoDSF: nanoscale differential scanning fluorimetry
NOESY: nuclear Overhauser effect spectroscopy
PUL: polysaccharide utilisation loci
*T*_m_
: melting temperature
TOCSY: total correlation spectroscopy
WEFT: water‐eliminated fourier transform
Δ: unsaturated 4‐deoxy‐α‐l‐threo‐hex‐4‐enopyranosiduronic acid

## Introduction

Macroalgae, or simply seaweeds, classified to brown (Phaeophyceae), red (Rhodophyta) and green (Chlorophyta) algae are significant photosynthetic organisms of the marine ecosystems, directly ensuring aquatic biomass production [1]. Brown (alginate) and red algae (carrageenan and agar) polysaccharides are widely exploited, mainly because of their hydrocolloid properties, while the green algae polysaccharide implementation potential is only sporadically investigated [2]. Green algae have previously been collected for food or feed needs; however, biomass exploitation for higher value products remains scarce, even though green algae are a proven source of compounds with beneficial properties including polysaccharides [3, 4].

**Fig. 1:**
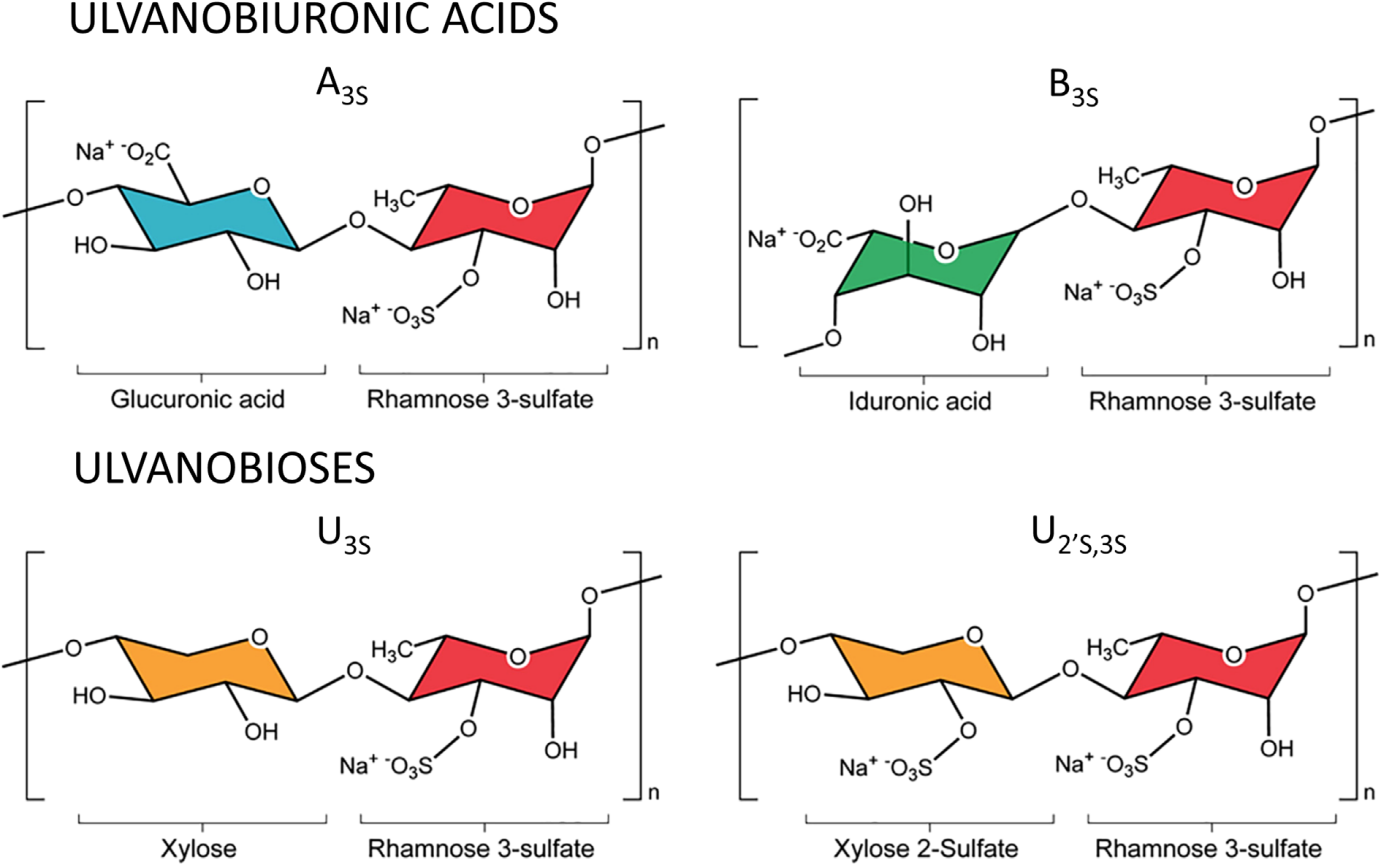
Nomenclature and structure of the major repeating disaccharide units that comprise ulvan polysaccharide [8]. Ulvanobiuronic acid A_3s_ contains glucuronic acid, outlined in blue, linked to rhamnose 3‐sulfate in red, while the similar B_3s_ contains rhamnose 3‐sulfate linked to iduronic acid in green, instead of glucuronic acid. Ulvanobioses are comprised of rhamnose 3‐sulfate linked to xylose in orange. Xylose can contain a sulfate group, as in U_2′s,3s_ disaccharide.

Ulvan is one of the most intriguing polysaccharides from green algae with prospected potential for food, material science, agricultural and pharmaceutical/medical application areas. These include its use as dietary fibre, building block of nanofibers or hydrogels, plant defence agent, antiviral and/or antimicrobial agent, antioxidant, anticoagulant, antihyperlipidemic agent, immunomodulant and antiproliferative agent against cancer cells, as well as a source of rare sugars for the chemical industry [5, 6]. Green seaweed, notably *Ulva* species, synthesise ulvan as a major cell wall structural polysaccharide, composing up to one third of the biomass dry weight [7]. Ulvan is a high molecular weight complex sulphated heteropolysaccharide composed of 3‐O‐sulfated α‐l‐rhamnose (α‐l‐Rha*p*3S), β‐d‐glucuronic acid (β‐d‐Glc*p*A), α‐l‐iduronic acid (α‐l‐Ido*p*A), β‐d‐xylose (β‐d‐Xyl*p*) and 2‐O‐sulfated β‐d‐xylose (β‐d‐Xyl*p*2S) residues [8]. Two major disaccharide aldobiuronic acid moieties (type A_3S_ [(→ 4)‐β‐d‐Glc*p*A‐(1 → 4)‐α‐l‐Rha3S‐(1 →)] and type B_3S_ [(→ 4)‐α‐l‐IdoA‐(1 → 4)‐α‐l‐Rha3S(1 →)] ulvanobiuronic‐3‐sulfates) connect with two minor disaccharide aldobiose moieties (type U_3S_ [(→ 4)‐β‐d‐Xyl*p*‐(1 → 4)‐α‐l‐Rha*p*3S‐(1 →)] and U_2′S,3S_ [(→ 4)‐β‐d‐Xyl*p*2S‐(1 → 4)‐α‐l‐Rha*p*3S‐(1 →)] ulvanobioses) in a repetitive manner forming a linear backbone structure of ulvan polysaccharide (Fig. 1) [6]. While β‐(1 → 4)‐glycosidic linkages are predominant in the ulvan backbone, β‐(1 → 2)‐ and β‐(1 → 3)‐glycosidic linkages also occur in the polysaccharide structure. The ulvan backbone is typically branched by β‐(1 → 2)‐linked β‐d‐Glc*p*A to A_3s_ ulvanobiuronic‐3‐sulfates [8]. The monosugar composition, sulphation as well as polysaccharide branching degree differ substantially depending on species and growth conditions leading to structural variety of ulvans [6]. Ulvan tends to be soluble in aqueous solutions; however, polysaccharide supramolecular aggregation to beads or gels of high viscosity is common [7]. Limitations of ulvan implementation due to high molecular weight, low solubility and comparatively low bioaccessibility could be overcome by applying enzymatic depolymerisation for ulvan oligosaccharide production [6]. Oligosaccharides produced from algal polysaccharides have demonstrated increased nutraceutical properties compared with intact polysaccharides [9].

Polysaccharide lyases (PL) (EC 4.2.2.‐) catalyse the initial cleavage of ulvan in an endo‐active manner targeting polysaccharide backbone β‐(1 → 4)‐glycosidic linkages between α‐l‐Rha*p*3S and β‐d‐Glc*p*A (type A_3S_) or between α‐l‐Rha*p*3S and α‐l‐Ido*p*A (type B_3S_) through β‐elimination with the formation of an unsaturated 4‐deoxy‐α‐l‐threo‐hex‐4‐enopyranosiduronic acid (Δ) at the nonreducing end of the reaction products [10]. Enzymatic degradation leads to ulvan depolymerisation into ulvan disaccharides (DP2) and/or ulvan tetrasaccharides‐(DP4), while DP4 could eventually be cleaved into DP2 [6]. Ulvan enzymatic depolymerisation has the advantages of degradation control, mild cleavage conditions and high product specificity.

Genes encoding ulvan lyases are usually annotated in polysaccharide utilisation loci (PUL) and were found in marine bacteria and marine animal gut bacteria, where they were proposed to act extracellularly to produce ulvan oligosaccharides that can be transported into cytoplasm for complete cleavage by various glycoside hydrolases [2]. Ulvan lyases with confirmed activity were attributed to PL24, PL25, PL28, PL37 and PL40 families [11]. Although a few PL24, PL25, and PL28 ulvan lyases were characterised, however most characterised ulvan lyases remain only sporadically investigated.

Ulvan lyases attributed to the PL25 family comprise a distinct group of lyases with distant sequence homologies to the PL24 family [12]. The relation between the PL25 and PL24 families is more apparent when comparing the two solved protein structures of PL24 (PDB 6BYP and PDB 7CZH) with the only PL25 structure solved (PDB 5UAM), where the enzymes from both families adapt a seven‐bladed β‐propeller fold with each propeller blade exhibiting four antiparallel β‐strands [13, 14]. Overall structure organisation formed by the different length blades includes a deep cleft on the upper surface of the propeller and neck at the entry to the cleft. Catalytic and substrate binding sites which involve His and Tyr combination acting as acid and base pair along with Arg acting as charge neutralizer ensuring β‐elimination catalytic mechanism are located in this cleft well‐defined in the PL25 family ulvan lyase structure [14]. The characterised PL25 lyases, sharing the three conserved catalytic residues, are encoded in genomes of closely related *Alteromonas*, *Pseudoalteromonas* and *Thalassomonas* strains as well as related *Algibacter luteus* and *Nonlabens ulvanivorans* type strains. Putative ulvan lyases attributable to the PL25 family were also annotated in numerous genomes of other Gram‐negative bacteria, while few sequences homologous to PL25 lyases were found even in archaea [11]. All nine currently characterised ulvan lyases attributed to the PL25 family, five of which originate from *Alteromonas* spp., catalyse ulvan oligosaccharide production from extracted ulvan polysaccharides. These enzymes were implemented for ulvan structure determination as well as acting solely or with glycoside hydrolases in processing of raw green algae biomass aiming to increase bioaccessibility of nutritious compounds [15, 16].

In this study, a novel ulvan lyase, termed SH2L_Ulv3, attributed to the PL25 family was characterised, phylogenetically distinct from previously characterised PL25 enzymes, expanding the armoury of available ulvan lyases for more efficient enzymatic depolymerisation of ulvan polysaccharides, aiding in green algae biomass exploitation. The characterised ulvan lyase was identified from a green and brown seaweed biomass metagenome enriched in an intertidal coastal hot spring [17]. The sequence of SH2L_Ulv3 was annotated as most similar to a hypothetical protein from a Bacteroidales bacterium identified from an aquatic metagenome [18]. This study provides an extensive biophysical and biochemical characterisation of SH2L_Ulv3 accompanied by a comprehensive bioinformatic analysis including three‐dimensional structure modelling and ligand docking simulations.

## Results

### SH2L_Ulv3 primary sequence analysis

Analysis of the presence of ulvan lyases encoded in a microbial metagenome extracted after *in situ* enrichment on green and brown seaweed biomass in the Skarðshver (SH) intertidal coastal hot spring located in Miðfjörður fjord (Iceland) led to identification of the *SH2L_Ulv3* gene (GenBank PP839856.1), from the large (L) sample bag (No. 2), annotated in the k141_543573 contig of 2297 bp length as ORF (GenBank XII33273.1) encoding a putative ulvan lyase, termed SH2L_Ulv3, attributable to PL25 family [17]. The sequence of SH2L_Ulv3 annotating was described as most similar (75% sequence identity over 71% query coverage) to a hypothetical protein DDX98_05900 fragment (GenBank HBH48151.1 [18]) from a bacterium identified from an aquatic metagenome classified under the order Bacteroidales.

The coding region of *SH2L_Ulv3* gene is 1362 bp with an overall GC‐content of 41.4%. The gene codes for a protein of 453 amino acids with an estimated isoelectric point at pH 5.73 and a calculated molecular mass of 50 512.77 Da. A signal peptide, ranging Met1‐Ser18, was predicted in the SH2L_Ulv3 sequence. The deduced amino acid sequence included predicted neuraminidase bacterial neuraminidase repeats (BNR)‐containing family member domain (BNR_4, Pfam PF15892), ranging Gly127‐Asn344, also described as superfamily sialidase (neuraminidase) superfamily region, ranging Lys86‐Lys258 (Sialidase_sf, InterPro IPR036278).

Comparison of the mature SH2L_Ulv3 sequence Cys19‐Glu453 with the signal peptide omitted revealed sequence homology to numerous hypothetical protein sequences with BNR_4 domain from mostly marine bacteria. Further analysis of sequence comparison, filtering homologous sequences of no < 50% identity to SH2L_Ulv3 sequence over 95–100% query coverage, resulted in a protein group containing 65 hypothetical proteins with BNR_4 domain. The SH2L_Ulv3 sequence within this group is most similar to the BNR_4 domain of a hypothetical BNR_4 two‐domain protein (RefSeq WP_045033608.1) with 64% identity over 99% query coverage, annotated in the draft genome of *Draconibacterium sediminis* JN14CK‐3^T^ [19], a bacterium classified under the order Marinilabiliales. Comparison of the SH2L_Ulv3 sequence with proteins from metagenomes revealed significant similarity with the hypothetical protein LCGC14_2758080 fragment (GenBank KKK86954.1 [20]) identified from a marine hydrothermal vents sediment metagenome with an observed 54% identity over 94% query coverage.

Comparison of the mature SH2L_Ulv3 sequence with structure determined proteins revealed homology, observing 45% identity over 97% query coverage, with the PL25 family PLSV_3936 ulvan lyase (RefSeq WP_033186995.1 [21]) from *Pseudoalteromonas* sp. PLSV. classified under the order Alteromonadales. Significant sequence similarities in the same range, with comparable 45–51% identity over 95–100% query coverage, was observed aligning the mature SH2L_Ulv3 sequence with characterised PL25 ulvan lyases (Table 1), what confirmed SH2L_Ulv3 attribution to PL25 family indicating some phylogenetic distance to the previously characterised ulvan lyases. Characterised PL25 ulvan lyases NLR_492 (IL45_03835) from *N. ulvanivorans* PLR [22] and ID172 from *A. luteus* P7‐3‐5 [16] 79% identical over 100% query coverage both from strains classified under the order Flavobacteriales were most similar to SH2L_Ulv3, while PLSV_3936 from *P*. sp. PLSV [14] and ULA‐3 from *Alteromonas* sp. 76‐1 [23] 77% identical over 99% query coverage both from strains classified under the order Alteromonadales shared lower sequence identity with the SH2L_Ulv3 sequence.

**Table 1:** SH2L_Ulv3 sequence Cys19‐Glu453 with signal peptide omitted comparison against sequences of characterised ulvan lyases attributed to PL25 family.

Ulvan lyase	Accession	Organism	References	Sequence^a^	Identity, %	Query, %
ALT3695 fragment	GenBank QFR04505.1	*Alteromonas* sp. A321	[28]	Pro1‐Lys444	49.42	95
LOR_29	RefSeq WP_052010178.1	*Alteromonas* sp. LOR	[22]	Cys34‐Leu492	47.00	96
NLR_492 (IL45_03835)	RefSeq WP_036580476.1	*Nonlabens ulvanivorans* PLR		Cys19‐Asp480	50.68	99
PLSV_3936	RefSeq WP_033186995.1	*Pseudoalteromonas* sp. PLSV	[14]	Cys32‐Lys496	44.62	97
TsUly25B	GenBank UKQ19338.2	*Thalassomonas* sp. LD5	[29]	Cys23‐Lys499	46.41	99
ULA‐3	RefSeq WP_129740764.1	*Alteromonas* sp. 76–1	[23]	Cys28‐Asn497	45.23	100
ULA‐2	RefSeq WP_131094886.1	*Alteromonas* sp. KUL42	[6]	Cys28‐Met492	47.58	96
ULA‐1	RefSeq WP_120963275.1	*Alteromonas* sp. TK‐46(2)	[25]	Met1‐Met472	47.93	96
ID172	RefSeq WP_050981764.1	*Algibacter luteus* P7‐3‐5	[16]	Cys17‐Glu480	50.23	97

^a^ Compared sequences after predicted signal peptide omission.

**Fig. 2:**
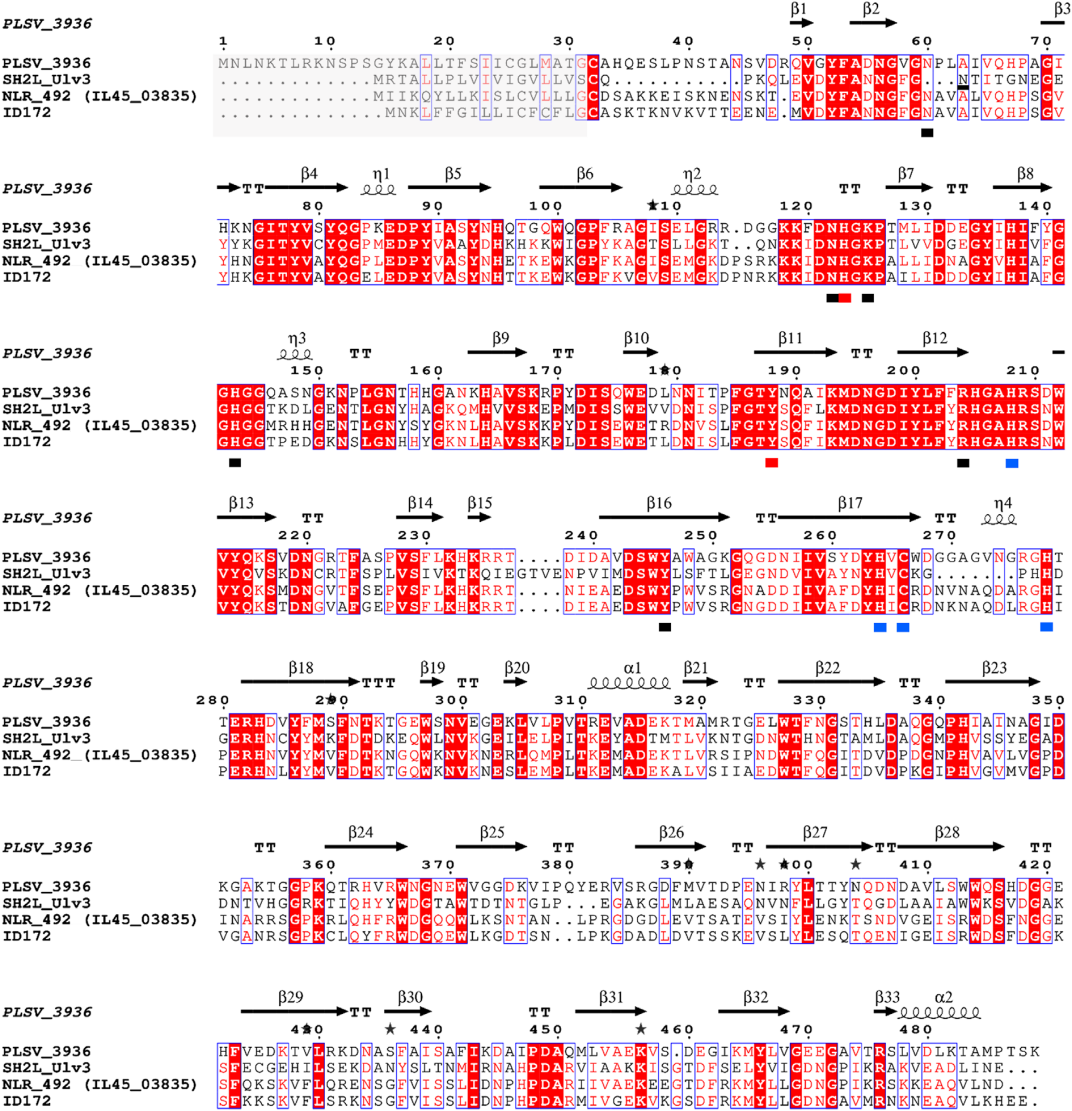
Multiple sequence alignment of SH2L_Ulv3 with PLSV_3936 ulvan lyase (RefSeq WP_033186995.1) from *Pseudoalteromonas* sp. PLSV [14], NLR_492 (IL45_03835) ulvan lyase (RefSeq WP_036580476.1) from *Nonlabens ulvanivorans* PLR [22] and ID172 ulvan lyase (RefSeq WP_050981764.1) from *Algibacter luteus* P7‐3‐5 [16] indicating conserved residues as well as secondary structure elements based on PLSV_3936 structure characterisation (PDB 5UAM [14]). Identical residues are presented in white and highlighted on a red background while similar residues are presented in red. TT = strict β turns. Signal peptides are highlighted in a grey background. Catalytic residues are underlined with red boxes, substrate binding residues are underlined with black boxes and zinc ion coordinating residues are underlined with blue boxes. The graphical representations of multiple sequence alignments were created with ESPript 3.0 server [41].

Alignment of the SH2L_Ulv3 sequence with the sequence of the only structure determined PL25 lyase PLSV_3936 (PDB 5UAM [14]) as well as with the most similar characterised PL25 lyases resulted in prediction of a catalytic module with conserved active and binding site residues (Fig. 2). The catalytic residues His95 and Tyr160 along with Arg176 predicted as comprising SH2L_Ulv3 active site corresponded to PLSV_3936 catalytic residues His123 and Tyr188 along with Arg204; meanwhile, three of the five substrate binding site residues Asn36, Asn94, Lys97, His115 and Tyr222 predicted in SH2L_Ulv3 sequence were corresponding to conserved Asn122, Lys125 and Tyr246 substrate binding residues in PLSV_3639 sequence (Fig. 2). Corresponding to His208, Cys266, His264 and His278 in PLSV_3936 sequence, Zn^2+^ ion coordinating residues were also predicted as His180, His240, Cys242 and His247 in the SH2L_Ulv3 sequence. Completely matching conserved active site residues and several conserved binding site residues for SH2L_Ulv3 supposes the catalytic mechanism as well as substrate binding and zinc ion coordination correspondence to suggested for the PL25 family PLSV_3936 ulvan lyase based on structure characterisation [14]. Secondary structure elements organisation characterised for PLSV_3936 was also predicted for SH2L_Ulv3 ulvan lyase (Fig. 2).

### SH2L_Ulv3 phylogenetic analysis

**Fig. 3:**
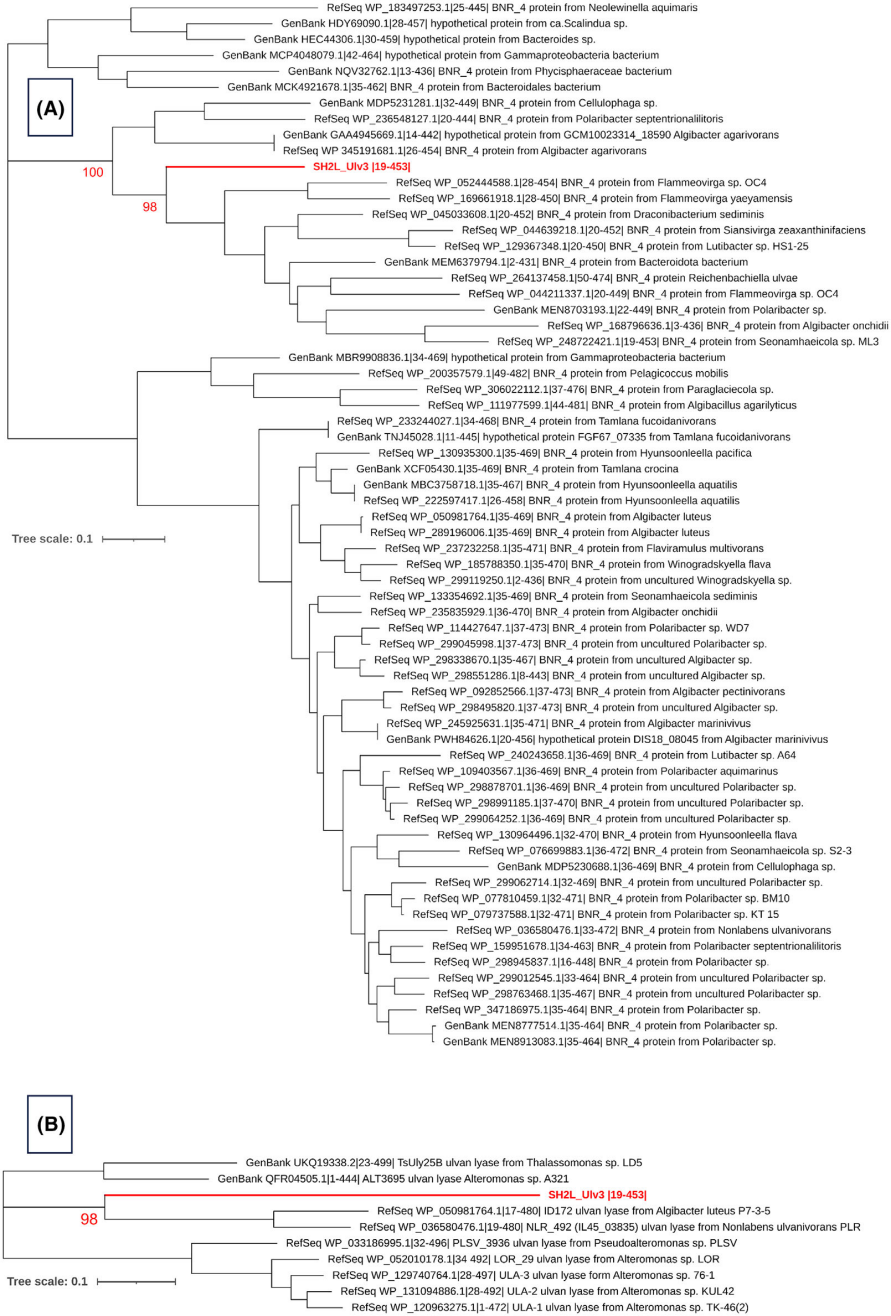
Phylogenetic analysis of SH2L_Ulv3 relationships, conducted with the most similar proteins as well as with characterised ulvan lyases attributed to PL25 family. (A) Phylogenetic tree calculated for SH2L_Ulv3 and the 65 protein sequences most similar to ulvan lyase. (B) Phylogenetic tree calculated for SH2L_Ulv3 and all nine characterised PL25 family ulvan lyase sequences. BNR_4, BNR repeat‐containing family member. The relevant bootstrap percent values are indicated at nodes. The mega11 suite [38] applying Jones–Taylor–Thornton matrix‐based model [39] was used to calculate maximum likelihood trees under default parameter values with 500 bootstrap replications [40]. ncbi blast suite output containing 65 most similar to SH2L_Ulv3 protein sequences accessible via nr database was extracted and re‐aligned after signal peptide omission and manual consideration with clustalw implemented in the mega11 suite under default parameter values for phylogenetic tree calculation. Phylogenetic tree analysing SH2L_Ulv3 relationships with characterised PL25 family ulvan lyases was generated analogously, extracting sequences accessible via CAZy database [11]. Graphical representations of the phylogenetic trees were created with itol v6 tool [42].

Phylogenetic tree calculation for the 65 protein sequences most similar to SH2L_Ulv3 grouped this ulvan lyase in a clade together with 15 hypothetical BNR_4 protein sequences with 100% bootstrap consensus (Fig. 3A). Further outgroup from 11 more diverged protein sequences for SH2L_Ulv3 was predicted with 98% support. The sequences not clustered in the clade with SH2L_Ulv3 were grouped with significant support into two independent clades inferring distant evolutionary relationships between predicted clades. The annotated hypothetical BNR_4 proteins that were evolutionary most related with SH2L_Ulv3 were mostly found in genomes of marine bacteria. The clade with SH2L_Ulv3 comprises hypothetical proteins, predominantly from Flavobacteriaceae bacteria, in particular from *Algibacter* strains, as well as proteins from Flammeovirgaceae bacteria, in particular from *Flammeovirga* strain genomes, and is also including the hypothetical BNR_4 protein from *D*. *sediminis* JN14CK‐3^T^. Interestingly, no other ulvan lyases are characterised among those phylogenetically more related sequences.

The characterised PL25 ulvan lyases from *Alteromonas* and *Pseudoalteromonas* strains were phylogenetically grouped together, except for the more distantly related ALT3696 ulvan lyase from *Alteromonas* sp. A321 that grouped with TsUly25B ulvan lyase from *Thalassomonas* sp. LD5 into an independent clade (Fig. 3B). The SH2L_Ulv3 sequence was clustered with 98% support inferring comparatively closer phylogenetic relationships with NLR_492 (IL45_03835) ulvan lyase from *N*. *ulvanivorans* PLR and ID172 ulvan lyase from *A*. *luteus* P7‐3‐5 into an independent clade. The sequences of characterised homologous ulvan lyases were notably less diverged compared with the SH2L_Ulv3 sequence.

### Recombinant protein production and purification

Cloned *SH2L_Ulv3* gene sequence was successfully expressed in *Escherichia coli* at excellent yield obtaining 18 mg of recombinant protein per litre of culture. Cloned site‐directed mutagenesis *SH2L_Ulv3‐His95Ala* and *SH2L_Ulv3‐Tyr160Ala* sequences were also successfully expressed in *E*. *coli* at excellent and moderate‐low yields of mutants, respectively (data not presented). Protein production had no toxic effect on expression culture growth. Recombinant proteins remained almost completely soluble in expression strain cells. Nickel affinity chromatography was successfully applied for protein purification to near homogeneity. The integrity and purity of proteins were successfully confirmed by SDS/PAGE. SH2L_Ulv3 and SH2L_Ulv3‐His95Ala mutants remained stable and non‐aggregation‐prone in formulation buffer, while aggregation of purified SH2L_Ulv3‐Tyr160Ala mutant was observed after storage at 4 °C.

### SH2L_Ulv3 molecular weight and oligomerisation

Oligomerisation analysed applying analytical size exclusion chromatography was not observed even at comparatively high SH2L_Ulv3 concentration. Pure SH2L_Ulv3 was detected as a single major peak of protein monomer, which remaining soluble in the analysis buffer (Fig. S1). The determined SH2L_Ulv3 elution volume almost completely corresponded to the expected elution volume for the calculated recombinant SH2L_Ulv3 monomer molecular mass of 49 624.34 Da (Fig. S1). Aggregation was not observed by applying analytical size exclusion chromatography, even after prolonged storage of SH2L_Ulv3 at 4 °C or freeze–thaw round after prolonged storage at −20 °C (data not presented).

### SH2L_Ulv3 activity and substrate specificity

**Fig. 4:**
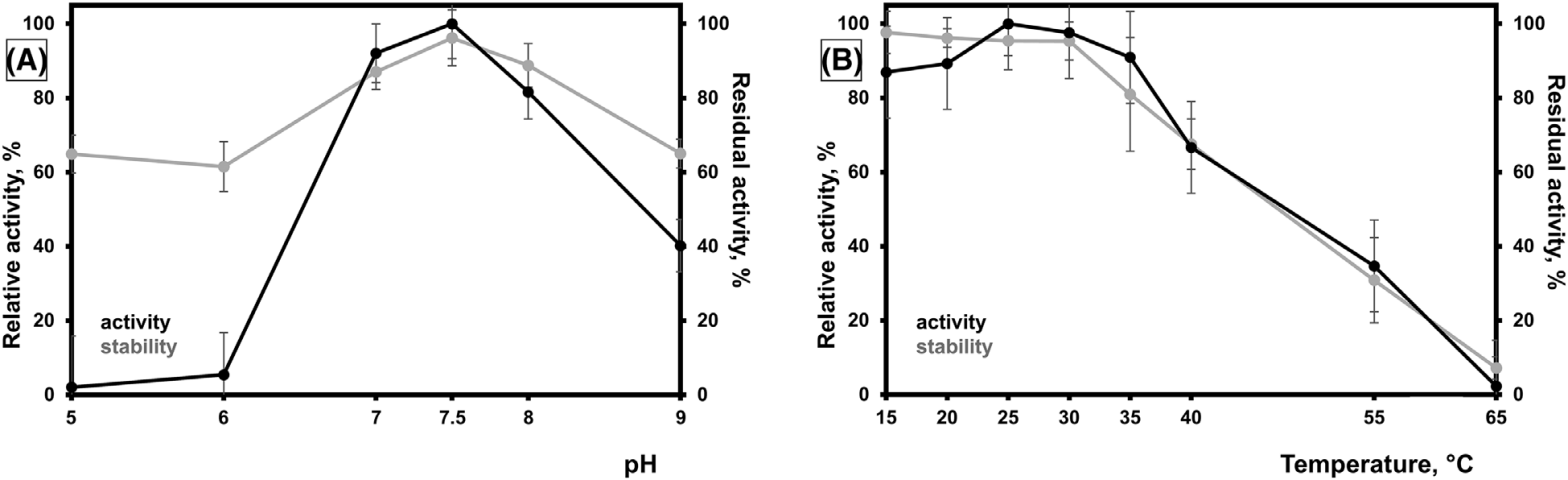
SH2L_Ulv3 activity optimum as well as pH stability and thermostability evaluation. (A) Influence of pH on SH2L_Ulv3 activity and stability. (B) Influence of temperature on SH2L_Ulv3 activity and stability. The pH optimum was determined in buffers (50 mm sodium acetate–acetic acid pH 5/RT, 50 mm MES‐NaOH pH 6/RT, 50 mm HEPES‐NaOH pH 7/RT and pH 7.5/RT, 50 mm Tris/HCl pH 8/RT as well as 50 mm glycine‐NaOH pH 9/RT). Enzyme ulvan from *Ulva armoricana* (fine grade) degrading activity at optimal pH (A) 7.56 ± 0.60 U·mg^−1^ or at optimal temperature (B) 8.04 ± 0.69 U·mg^−1^, respectively, was defined as 100%. All measurements were performed in triplicates. Error bars represent standard deviation.

Ulvan from *Ulva armoricana* (fine grade) was used as substrate evaluating SH2L_Ulv3 activity optimum as well as pH stability and thermostability. SH2L_Ulv3 activity was determined over a pH range of pH 5–9 measured at 30 °C (Fig. 4A). Purified SH2L_Ulv3 ulvan lyase was barely active at pH 6 and 5 with activity not exceeding 6% (Fig. 4A), and no activity was detected at pH 4 (data not presented). Over the narrow pH 7–8 range, the highest SH2L_Ulv3 activity was detected exceeding 80% of the maximum activity with a comparatively blunt pH optimum of pH 7.5 at 30 °C. A shift towards a more alkaline pH range was further observed as a more gradual activity decrease over alkaline pH with the enzyme activity at pH 9 reaching approximately 40% of the maximum activity. Activity determination of SH2L_Ulv3 at different temperatures at pH 7.5 resulted in an activity plateau with < 15% difference over a temperature range of 15–35 °C (Fig. 4B), with an apparent temperature optimum at 25 °C. However, the purified enzyme was effectively active in the temperature range of 35–55 °C, with relative activity at 40 °C exceeding 66% of the maximum activity and at 55 °C exceeding 34%. Gradual activity decline continued in the temperature range of 55–65 °C, and SH2L_Ulv3 activity was < 3% of the maximum at 65 °C. SH2L_Ulv3 demonstrated pH stability over a pH range of pH 5–9 with determined residual activity exceeding 60% (Fig. 4A). Incubation for 30 min at pH 7–8 had no significant influence on SH2L_Ulv3 activity, while comparatively similar residual activity decrease reaching 40% was observed at pH 5–6 and at pH 9. SH2L_Ulv3 remained thermostable for 30 min at the temperature range of 15–35 °C almost completely retaining ulvan degrading activity (Fig. 4B), while characterised ulvan lyase was gradually losing activity after preincubation at 40–65 °C. Residual activity after preincubation at 40 °C, 55 °C and 65 °C was reaching 68%, 31% and 7%, respectively. SH2L_Ulv3 activity was completely inactivated by preincubation at 95 °C for 10 min.

**Fig. 5:**
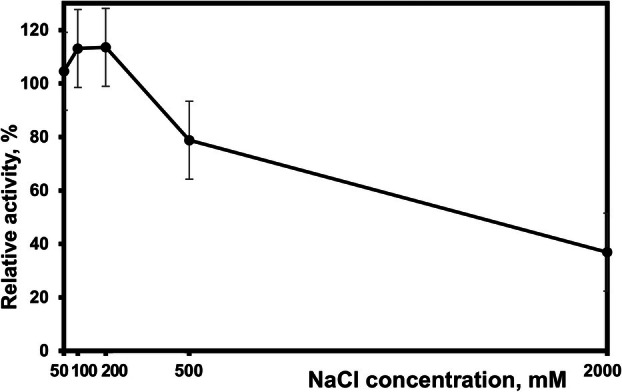
Influence of NaCl concentration on SH2L_Ulv3 activity. Enzyme ulvan from *Ulva armoricana* (fine grade) degrading activity at optimal pH and temperature 8.28 ± 0.37 U·mg^−1^ was defined as 100%. All measurements were performed in triplicates. Error bars represent standard deviation.

Influence of divalent metal ions on SH2L_Ulv3 activity evaluation determined that only Mg^2+^ ion had a slight stimulating effect on enzyme activity significant determining metal ion influence at 1 mm concentration (Table 2), while 10 mm (Table 2) or 50 mm (data not presented) Mg^2+^ ion concentrations had no effect on activity. SH2L_Ulv3 activity was not influenced by Ca^2+^ and Mn^2+^ ion evaluated concentrations. Co^2+^ ion significantly reduced the relative activity to 40%, Ni^2+^ reduced the activity to 20% of the initial, and only 8% of the activity remained after the 30 min preincubation with Cu^2+^ ion. Influence of Co^2+^, Ni^2+^ and Cu^2+^ ions both evaluated concentrations on SH2L_Ulv3 activity did not differ significantly. Nonlinear concentration dependent Fe^2+^ and Zn^2+^ ion influence reducing enzyme activity was determined. Zn^2+^ reduced relative activity to 20% and 8% at 1 and 10 mm concentrations, respectively. Fe^2+^ ion at 10 mm reduced activity to 40%, while 1 mm concentration reduced relative activity by an additional 10–30% (Table 2). Chelation with 1 or 10 mm EDTA serving as a corresponding control had little effect on SH2L_Ulv3 activity. Influence of NaCl concentration on SH2L_Ulv3 activity determined 200 mm NaCl as optimal for enzyme activity (Fig. 5). Both 100 and 200 mm NaCl increased SH2L_Ulv3 ulvan degrading activity by at least 10%. No significant influence on SH2L_Ulv3 activity was determined supplementing activity reaction with 50 mm NaCl, and SH2L_Ulv3 activity was reduced by concentrations exceeding 200 mm NaCl. At 500 and subsequent 2000 mm NaCl activity decreased by 20% and 70% of relative activity, respectively. No significant synergistic effect of 10 mm CaCl_2_ and 200 mm NaCl on SH2L_Ulv3 activity was observed, and supplementation of activity reaction with 50 mm KCl, also evaluated, had no significant effect on the enzyme activity (data not presented).

**Table 2:** Influence of divalent metal ions on SH2L_Ulv3 activity.

Metal ion	1 mm ion concentration	10 mm ion concentration
	Relative activity^a^, ^b^ ± standard deviation, %	
EDTA, control	99.52 ± 2.43	92.17 ± 6.64
Mg^2+^	106.44 ± 2.44	103.77 ± 8.85
Ca^2+^	102.55 ± 1.89	92.33 ± 3.77
Mn^2+^	99.90 ± 2.61	95.69 ± 8.66
Fe^2+^	29.43 ± 4.12	42.27 ± 2.01
Co^2+^	40.63 ± 5.74	39.16 ± 7.75
Ni^2+^	27.60 ± 5.96	18.95 ± 2.73
Cu^2+^	12.37 ± 8.30	7.57 ± 5.12
Zn^2+^	24.06 ± 7.56	8.23 ± 4.19

^a^ Enzyme ulvan from *Ulva armoricana* (fine grade) degrading activity at optimal pH and temperature 8.28 ± 0.37 U·mg^−1^ was defined as 100%.

^b^ Measurements were performed in triplicates.

**Fig. 6:**
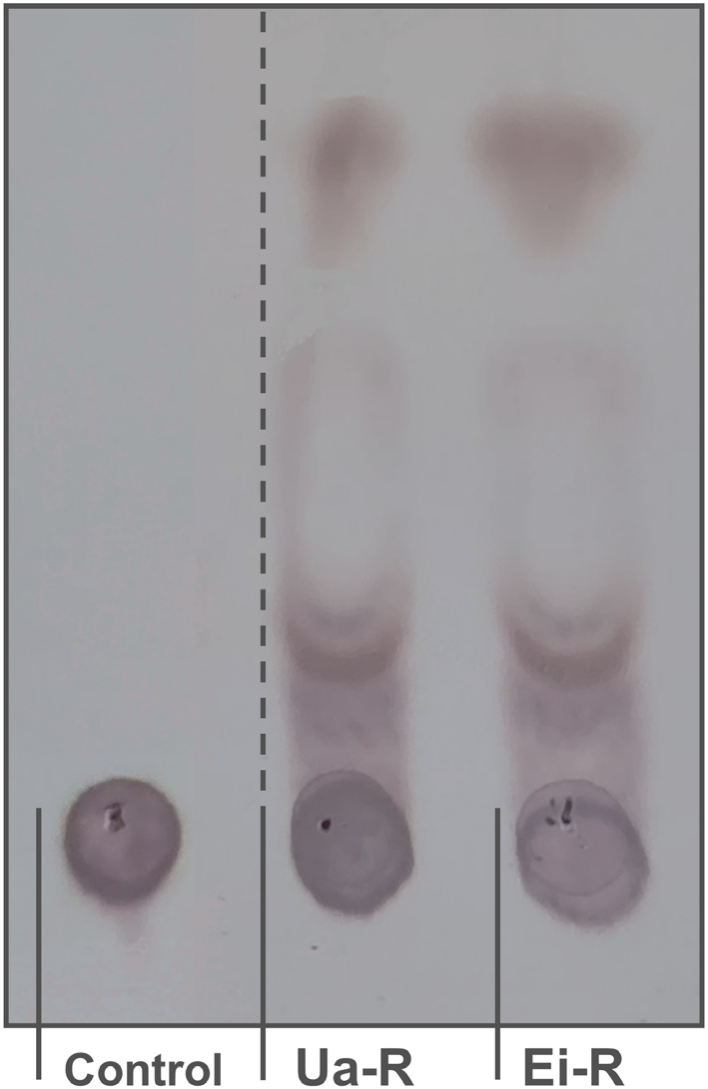
SH2L_Ulv3 reaction of ulvan degradation product prolife TLC visualisation. (Control) Ulvan from *Ulva armoricana* (fine grade); (Ua‐R) Optimal activity reaction with ulvan from *U. armoricana* fractionation; (Ei‐R) Optimal activity reaction with ulvan from *Enteromorpha intestinalis* (fine grade) fractionation. The vertical dashed line indicates where lanes have been spliced together.

SH2L_Ulv3 effectively degraded ulvan from both *U*. *armoricana* (fine grade) and *Enteromorpha intestinalis* (fine grade) performing optimal ulvan degrading activity reaction. Degradation product profiles of evaluated substrates fully corresponded (Fig. 6). SH2L_Ulv3 degraded ulvan from *U*. *armoricana* (fine grade) at specific activity 9.52 U·mg^−1^, while ulvan from *E*. *intestinalis* (fine grade) was degraded nine times less efficiently at only 0.86 U·mg^−1^ performing activity reaction incubation for 1 h (Table 3) what indirectly indicated structural and/or compositional differences of the used polysaccharides. Evaluating substrate specificity at prolonged reaction incubations specificity preference of ulvan from *U*. *armoricana* (fine grade) by characterised ulvan lyase remained obvious, even though at prolonged activity reaction incubations ulvan from *E. intestinalis* (fine grade) depolymerisation was three times less efficient than evaluated for optimal SH2L_Ulv3 substrate.

**Table 3:** SH2L_Ulv3 substrate specificity.

Reaction^a^ time, h	0.5% (w/v) ulvan from *Ulva armoricana* (fine grade)	0.5% (w/v) ulvan from *Enteromorpha intestinalis* (fine grade)
	Specific activity^b^ ± standard deviation, U·mg^−1^	
1	09.52 ± 0.09	00.86 ± 0.23
3	07.39 ± 0.06	02.17 ± 0.14
24	15.52 ± 0.03	03.33 ± 0.12
48	15.66 ± 0.31	04.10 ± 0.74

^a^ Enzyme degrading activity reaction at optimal pH and temperature in optimal reaction buffer (50 mm HEPES–NaOH pH 7.5/RT and 200 mm NaCl).

^b^ Measurements were performed in triplicates.

### SH2L_Ulv3 thermostability

SH2L_Ulv3 thermostability was estimated in the formulation buffer containing 10% (v/v) glycerol determining the melting temperature (*T*_m_) applying nanoscale differential scanning fluorimetry (nanoDSF) in the absence and presence of ulvan polysaccharide. All measurements resulted in a well‐defined single transition peak confirming that presence of substrate increase the apparent unfolding temperature. Thermostability reaching *T*_m_ 42.07 ± 0.06 and 42.17 ± 0.05 °C was determined for SH2L_Ulv3 without substrate at 0.1 and 0.2 mg·mL^−1^ protein concentrations, respectively, whereas *T*_m_ 43.53 ± 0.12 and 47.13 ± 0.31 °C were determined for SH2L_Ulv3 at a concentration of 0.1 mg·mL^−1^ with 0.05 and 0.5% (w/v) ulvan from *U*. *armoricana* (fine grade), respectively. SH2L_Ulv3 was significantly more thermostable by *T*_m_ 5 °C in presence of 0.5% (w/v) substrate and even comparatively low 0.05% (w/v) substrate concentration ensured thermal unfolding temperature increase by *T*_m_ 1.5 °C. Presence of the ulvan polysaccharide also increased SH2L_Ulv3 transition start temperature measured at 32–33 °C to 34 °C measured with 0.05% (w/v) and 39 °C with 0.5% (w/v) ulvan. Comparison of unfolding temperatures revealed minor influence of protein concentration, however significant stabilisation of the folded enzyme by substrate. SH2L_Ulv3 ulvan lyase is not thermostable at temperature higher than 42 °C without substrate and 47 °C with substrate.

### SH2L_Ulv3 kinetics

SH2L_Ulv3 kinetic parameters were determined by performing optimal activity reaction with different concentrations of ulvan from *U*. *armoricana* (fine grade) used as optimal substrate were *K*_M_ 3.63 ± 0.12 mg·mL^−1^, *V*_max_ 1.78 ± 0.04 μmol·min^−1^·mL^−1^ and subsequently *k*_cat_ 1.46 ± 0.04 s^−1^.

### SH2L_Ulv3 site‐directed mutagenesis

Site‐directed mutation of putative catalytic amino acid residues His95 or Tyr160 to Ala completely inactivated SH2L_Ulv3 ulvan lyase as SH2L_Ulv3‐His95Ala and SH2L_Ulv3‐Tyr160Ala mutants demonstrated no catalytic activity performing optimal activity reaction using optimal substrate.

### Zinc content in SH2L_Ulv3 sample

Measured applying inductively coupled plasma optical emission spectroscopy (ICP‐OES) zinc concentration in pure SH2L_Ulv3 sample was 0.55 ± 0.03 mg·L^−1^ what corresponded to molar protein:zinc ratio 1 : 0.52 as pure protein amount in quantified samples was 8 mg of recombinant SH2L_Ulv3.

### Ulvan degradation products

The oligosaccharide fraction from SH2L_Ulv3 optimal reaction using ulvan from *U. armoricana* (fine grade) performed at up to 24 h prolonged incubation was purified applying preparative size exclusion chromatography (Fig. S2A). Intact as well as partially enzymatically degraded ulvan and oligosaccharide fractions were separated at comparatively high resolution by preparative chromatography, as confirmed by TLC visualisation, however, further separation of oligosaccharide fraction was needed to obtain individual ulvan oligosaccharides. Size exclusion chromatography was further applied for oligosaccharide separation at high resolution to obtain adequate purity and amounts of the four most abundant individual ulvan oligosaccharides for identification applying NMR spectroscopy.

The four fractions containing individual oligosaccharides were subjected to NMR spectroscopy. ^1^H and ^13^C NMR resonances were assigned from total correlation spectroscopy (TOCSY), nuclear Overhauser effect spectroscopy (NOESY), heteronuclear multiple bond correlation (HMBC), and heteronuclear single quantum coherence (HSQC) experiments, and subsequently compared for structure identification of ulvan degradation products [24].

**Fig. 7:**
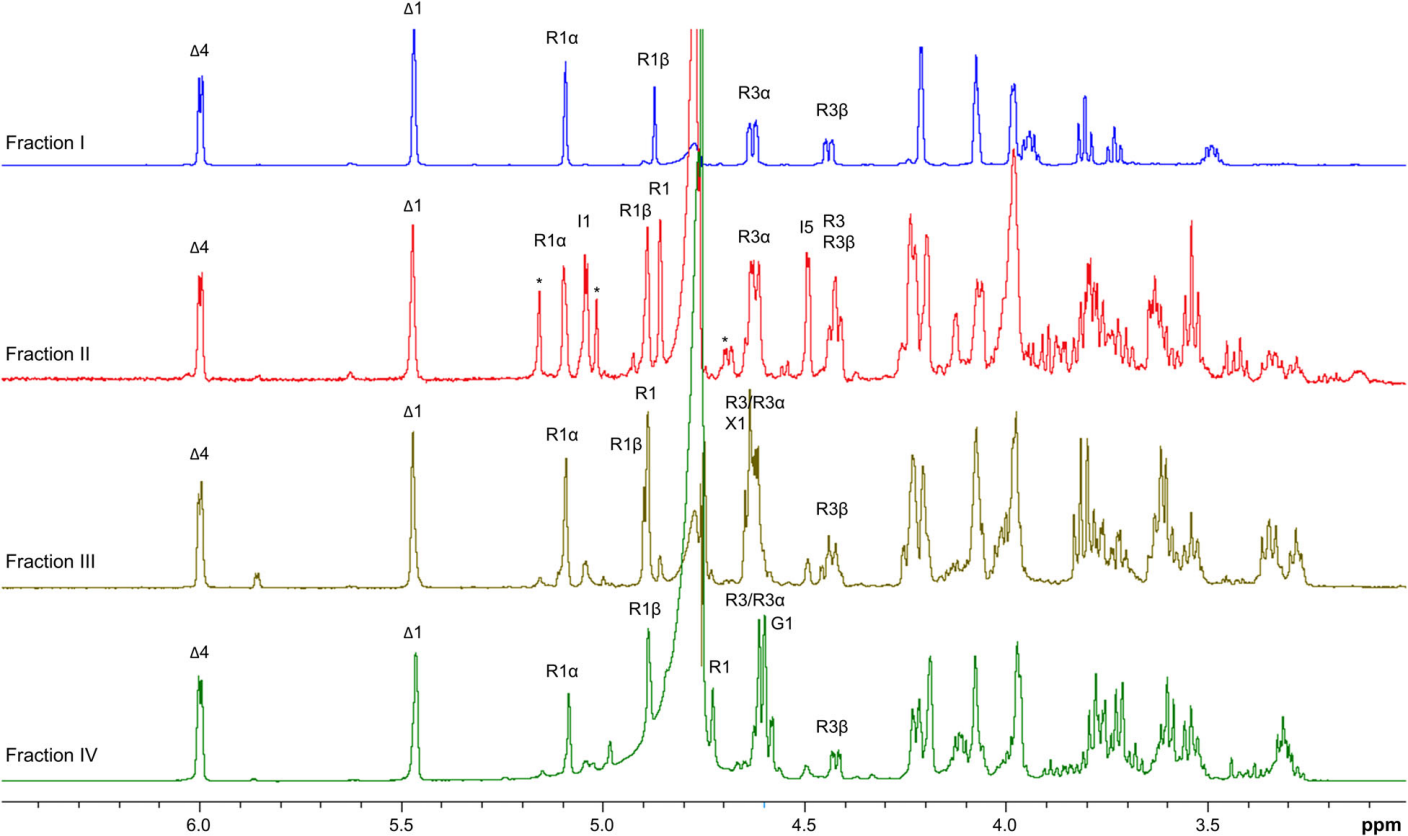
1D ^1^H NMR spectra of four individual ulvan oligosaccharide samples recorded in D_2_O at 298 K. Oligosaccharides were separated from oligosaccharide fraction purified from SH2L_Ulv3 optimal reaction with ulvan from *Ulva armoricana* (fine grade) performed at prolonged up to 24‐h incubation. G, β‐d‐Glc*p*A; I, α‐l‐Ido*p*A; R, α‐l‐Rha*p* 3‐sulfate; Rα/β, α‐ or β‐l‐Rha*p* 3‐sulfate reducing ends; X, β‐d‐Xyl*p*; Δ, 4‐deoxy‐α‐l‐threo‐hex‐4‐enopyranosiduronic acid at the nonreducing end. *Minor quantities of other oligosaccharides.

The 1D ^1^H NMR spectrum of fraction I sample (Fig. 7) demonstrated anomeric signals at δ_H_ 5.11 (Rα) and δ_H_ 4.89 (Rβ), both attributed to a reducing end of l‐Rha*p*3S residue. Nonreducing end Δ residue, through a β‐elimination formed from β‐d‐Glc*p*A and/or α‐l‐Ido*p*A residues during ulvan depolymerisation, was traced by its H‐4 (Δ) signal observed at δ_H_ 6.01. The assignments of the ^1^H and ^13^C resonances of fraction I were performed by means of 2D TOCSY and 2D HSQC experiments (Table 4). The downfield δ of l‐Rha*p*3Sα/β residue (δ_C‐4_ 77.6 (Rα)/77.4 (Rβ)) in fraction I identifies disaccharide composed of nonreducing Δ (1 → 4)‐linked to l‐Rha*p*3S (Table 4, Fig. S3).

**Table 4:** ^1^H and ^13^C NMR chemical shifts (δ) of separated SH2L_Ulv3 ulvan lyase reaction oligosaccharide fraction ulvan oligosaccharides. ND, not determined.

Residue	H‐1	H‐2	H‐3	H‐4	H‐5	CH_3_
	C‐1	C‐2	C‐3	C‐4	C‐5	
Disaccharide: ∆‐(1 → 4)‐l‐Rha*p*3Sα/β					Fraction I	
Rα	5.11	4.22	4.64	3.82	3.95	1.16
→ 4)‐l‐Rha*p*3Sα	94.8	70.8	80.1	77.6	68.2	18.0
Rβ	4.89	4.22	4.45	3.74	3.50	1.16
→ 4)‐l‐Rha*p*3Sβ	94.3	71.0	81.9	77.4	71.6	18.0
**∆**	5.48	4.08	3.99	6.01	–	–
∆‐(1 →	100.3	69.1	65.3	107.5		
Tetrasaccharide: ∆‐(1 → 4)‐α‐l‐Rha*p*3S‐(1 → 4)‐α‐l‐Ido*p*A‐(1 → 4)‐l‐Rha*p*3Sα/β					Fraction II	
Rα	5.11	4.25	4.64	3.83	4.00	1.27
→ 4)‐l‐Rha*p*3Sα	94.7	70.6	79.8	77.5	68.7	17.7
Rβ	4.91	4.25	4.43	ND	3.54	1.32
→ 4)‐l‐Rha*p*3Sβ	94.3	70.6	79.7		71.4	18.4
I	5.06	3.65	3.81	3.99	4.51	–
→ 4)‐α‐l‐Ido*p*A‐(1 →	104.2	71.9	72.8	80.7	72.2	
R	4.87	4.21	4.43	3.99	4.01	1.16
→ 4)‐α‐l‐Rha*p*3S‐(1 →	102.4	69.9	79.7	80.7	68.7	17.8
∆	5.49	4.08	4.00	6.01	–	–
∆‐(1 →	100.3	69.1	65.3	107.6		
Tetrasaccharide: ∆‐(1 → 4)‐α‐l‐Rha*p*3S‐(1 → 4)‐β‐d‐Xyl*p*‐(1 → 4)‐l‐Rha*p*3Sα/β					Fraction III	
Rα	5.11	4.25	4.64	3.81	3.99	1.32
→ 4)‐l‐Rha*p*3Sα	94.7	70.4	79.4	78.6	68.7	18.4
Rβ	4.91	4.26	4.46	3.73	3.55	1.33
→ 4)‐l‐Rha*p*3Sβ	94.3	71.1	81.4	77.7	71.5	18.4
X	4.65	3.29	3.62	3.64	3.37, 4.08	–
→ 4)‐β‐d‐Xyl*p*‐(1 →	104.8	74.6	74.7	75.1	63.5	
R	4.91	4.22	4.64	3.83	4.03	1.16
→ 4)‐α‐l‐Rha*p*3S‐(1 →	98.8	70.1	80.1	77.6	68.7	17.8
∆	5.49	4.09	3.99	6.01	–	–
∆‐(1 →	100.4	69.1	65.4	107.6		
Tetrasaccharide: ∆‐(1 → 4)‐α‐l‐Rha*p*3S‐(1 → 4)‐β‐d‐Glc*p*A‐(1 → 4)‐l‐Rha*p*3Sα/β					Fraction IV	
Rα	5.11	4.25	4.63	3.78	4.01	1.23
→ 4)‐l‐Rha*p*3Sα	94.6	70.2	79.5	79.5	68.8	18.3
Rβ	4.91	4.26	4.45	3.70	3.54	1.33
→ 4)‐l‐Rha*p*3Sβ	94.3	70.6	81.2	79.0	72.2	18.3
G	4.63	3.33	3.63	3.57	3.74	–
→ 4)‐β‐d‐Glc*p*A‐(1 →	104.4	74.9	75.0	80.1	77.3	176.5
R	4.75	4.21	4.61	3.80	4.14	1.14
→ 4)‐α‐l‐Rha*p*3S‐(1 →	101.1	70.1	79.9	77.5	68.6	17.6
∆	5.49	4.10	3.99	6.02	–	–
∆‐(1 →	100.3	69.1	65.3	107.6		

The 1D ^1^H NMR spectrum of fraction II sample demonstrated five major anomeric signals (Fig. 7), correlating with Δ, Rha3S (R), Ido*p*A (I) and Rha*p*3Sα/β (Rα/Rβ) residues. The anomeric region looks similar to corresponding region of fraction I, with the exception of two extra anomeric signals observed at δ_H_ 5.06 (I) and δ_H_ 4.87 (R), respectively (Fig. 7). The ^1^H δ of the remaining anomeric signals at δ_H_ 5.49 (Δ), δ_H_ 5.11 (Rα), δ_H_ 4.91 (Rβ) corresponds to δ found in fraction I spectrum. The HSQC spectrum demonstrated the downfield shifts at δ_C‐4_ 80.7 (I), δ_C‐4_ 80.7 (R) and at δ_C‐4_ 77.5 (Rα), indicating the involvement of these carbons in glycosidic linkages (Table 4, Fig. S4). The inter‐residue NOESY cross peaks (data not presented) between H‐1 (Δ) at δ_H_ 5.49 and H‐4 (R) at δ_H_ 3.99, H‐1 (R) at δ_H_ 4.87 and H‐4 (I) δ_H_ 3.99, H‐1 (I) at δ_H_ 5.06 and H‐4 (Rα) at δ_H_ 3.82 confirmed that all residues were (1 → 4)‐linked into tetrasaccharide ∆‐(1 → 4)‐α‐l‐Rha*p*3S‐(1 → 4)‐α‐l‐Ido*p*A‐(1 → 4)‐l‐Rha*p*3Sα/β.

The 1D ^1^H NMR spectra of fraction III and fraction IV samples (Fig. 7) were identical to tetrasaccharides ∆‐(1 → 4)‐α‐l‐Rha*p*3S‐(1 → 4)‐β‐d‐Xyl*p*‐(1 → 4)‐l‐Rha*p*3Sα/β and ∆‐(1 → 4)‐α‐l‐Rha*p*3S‐(1 → 4)‐β‐d‐Glc*p*A‐(1 → 4)‐l‐Rha*p*3Sα/β [24]. The signal corresponding to the anomeric proton of Xyl (X) residue was observed at δ_H_ 4.65, whereas the anomeric proton attributed to Glc*p*A was observed at δ_H_ 4.63. The GlcA (G) residue was distinguished from the Xyl residue by the distinct anomeric proton position of the neighbouring Rha3S (R) residue (δ_H_ 4.75 (R–G) and δ_H_ 4.91 (R–X)). The remaining anomeric signals were identified as Δ (δ_H_ 5.49), Rα (δ_H_ 5.11) and Rβ (δ_H_ 4.91) residues, respectively (Table 4, Figs S5 and S6).

DP2 and three DP4 were identified as the oligosaccharide products of the SH2L_Ulv3 reaction (Fig. S2B) determining DP2 as prevailing product in oligosaccharide fraction of ulvan degradation by characterised ulvan lyase.

### Enzyme structure modelling and ligand docking simulations

**Fig. 8:**
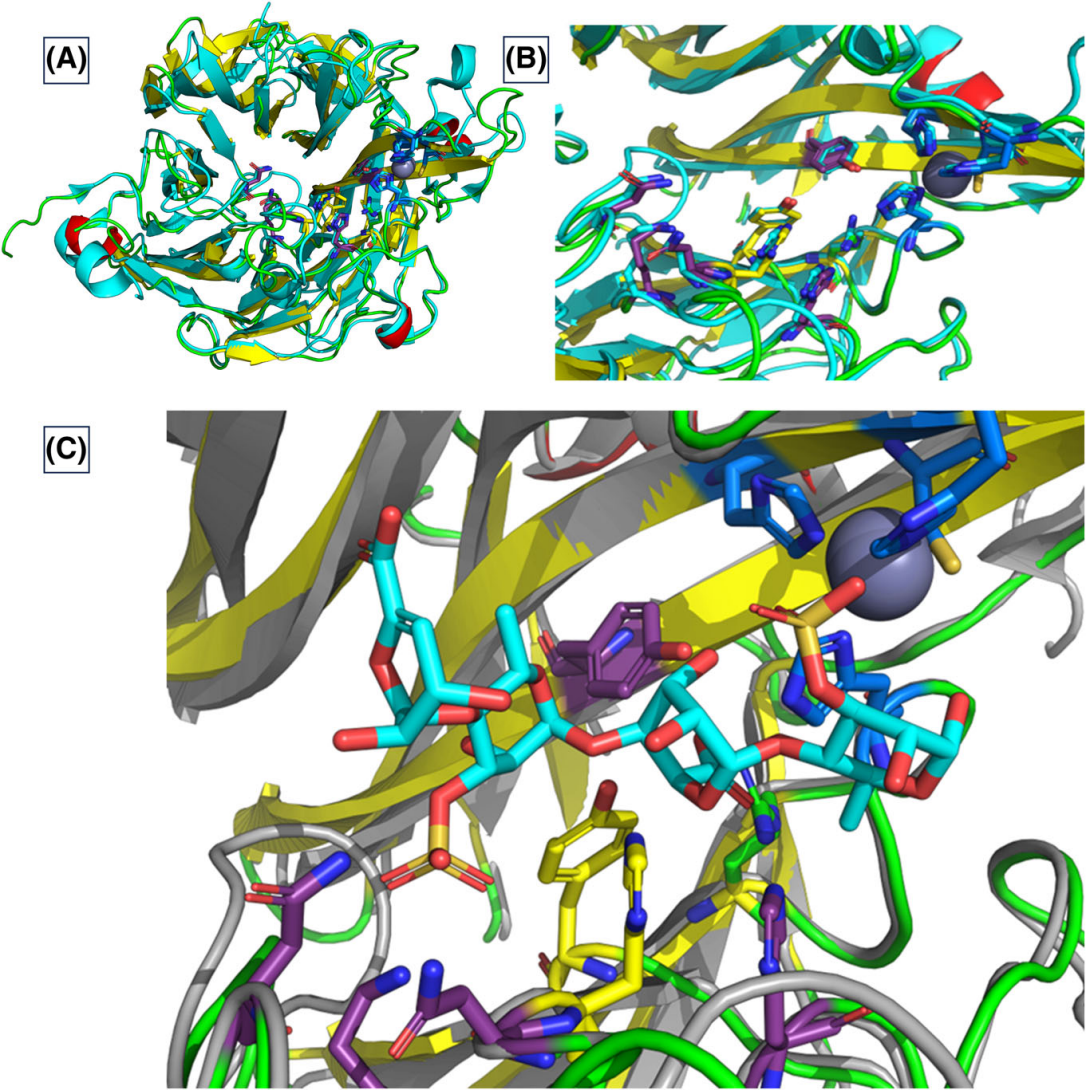
SH2L_Ulv3 model active site structure alignment with PLSV_3936 ulvan lyase from *Pseudoalteromonas* sp. PLSV active site [14]. (A) SH2L_Ulv3 superimposed with PDB 5UAM chain B structure highlighting active site residues. (B) SH2L_Ulv3 and PDB 5UAM chain B structure active site superimposition. (C) SH2L_Ulv3 and PDB 5UAS chain B including ∆‐(1 → 4)‐α‐l‐Rhap3S‐(1 → 4)‐β‐d‐GlcpA‐(1 → 4)‐α‐l‐Rhap3S as a ligand structure active site superimposition. Zn^2+^ is presented in dark grey. The catalytic residues are presented in yellow, metal ion coordinating residues in blue and substrate binding residues in purple. Structure ligand is coloured by element with carbon in cyan. Structural modelling was conducted with the AlphaFold3 model [47]. Graphical visualisations of the structure models, as well as structure superimpositions, were created with the pymol 3.1 system (Schrödinger).

The SH2L_Ulv3 ulvan lyase AlphaFold3 structural model revealed topology as well as overall fold similarity to the solved structure of PLSV_3936 ulvan lyase from *P*. sp. PLSV (PDB 5UAM and PDB 5UAS [14]). The structural similarity of SH2L_Ulv3 structural model to the experimentally determined structure of PLSV_3936 ulvan lyase was expected considering the high sequence similarity. The overall structural organisation of both lyases adopts a seven‐bladed β‐propeller fold including a deep cleft on the surface of the propeller lined by the antiparallel β‐strands and a neck at the entry to the cleft (Fig. S7A,B). The SH2L_Ulv3 β‐propeller fold; each propeller blade is formed up of 3–4 β strands as in the PLSV_3936 ulvan lyase. The structural alignment confirmed very high similarity as the SH2L_Ulv3 model superimposed with PDB 5UAM chain B and PDB 5UAS chain B structures with RMSD measures of 0.903 and 0.922 Å, respectively. Location as well as coordination geometry of the structural Zn^2+^ ion in the model SH2L_Ulv3 structure was identical to the location observed in 5UAM and 5UAS structures. The zinc ion was coordinated by residues His180, His240, Cys242 and His247 in a tetrahedral coordination geometry and located 10.0 and 9.3 Å from the proposed catalytic residues His95 and Tyr160, respectively, comparatively near to the neck of the structural cleft. The SH2L_Ulv3 catalytic, zinc ion coordinating as well as conserved substrate binding residues (Fig. 2) positioning were thus completely congruent with the positions of the corresponding residues in the structural comparison of the enzyme model with PDB 5UAM and PDB 5UAS structures (Fig. 8A,B). The ulvan tetrasaccharide substrate bound as ligand to PLSV_3936 ulvan lyase structure PDB 5UAS [14] superimposed on SH2L_Ulv3 model (Fig. 8C) placed the ligand directly in the active site with the scissile bond (between the sulphated rhamnose subsite −1 and the glucuronic acid subsite +1) located appropriately for cleavage with 2.6 Å distances from the C5 of the reacting uronic acid (glucuronic acid in the ulvan tetrasaccharide) to the closest His95N^τ^ and with 3.0 Å distance from the oxygen in the scissile bond to Tyr160O, with the carboxylic group orientated in the Asn36 direction for charge neutralisation. The substrate binding correspondence in SH2L_Ulv3 model supposes the active site architecture able to ensure β‐elimination confirming catalytic mechanism expected for a polysaccharide lyase.

**Fig. 9:**
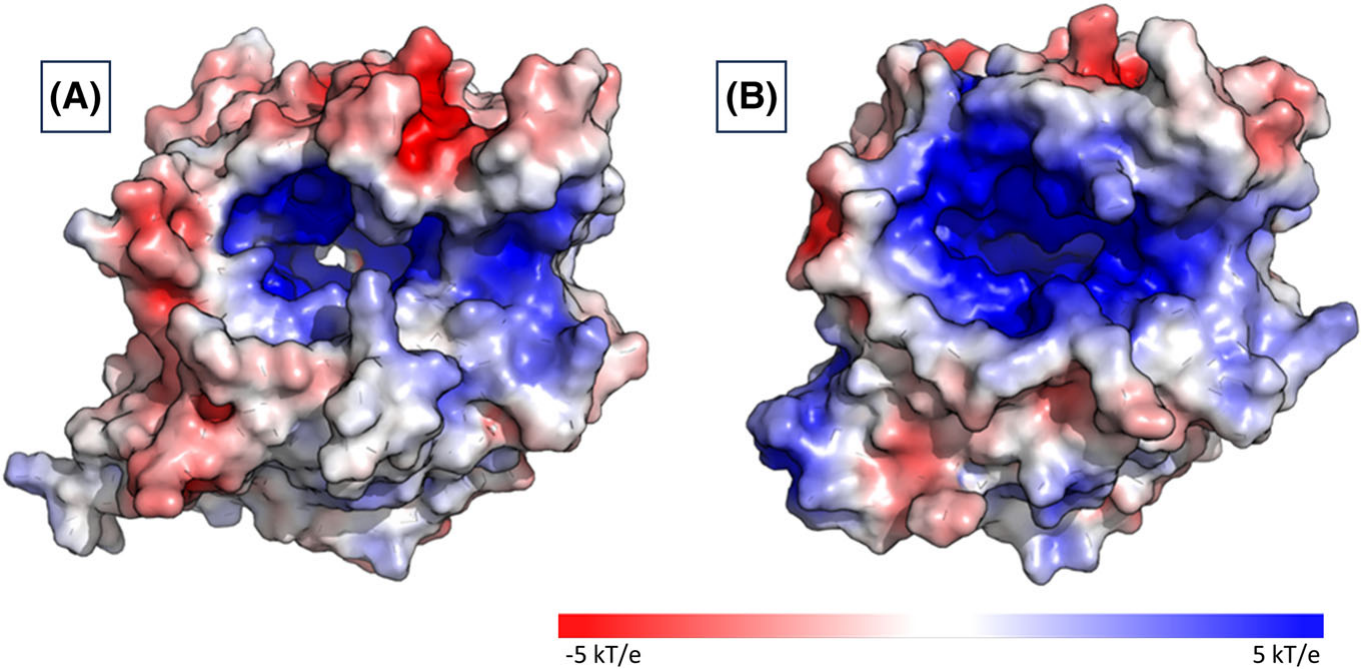
The electrostatic surface potential of SH2L_Ulv3 structure model and PLSV_3936 ulvan comparison. (A) SH2L_Ulv3 structure model. (B) PLSV_3936 ulvan lyase (PDB 5UAM [14]). The electrostatic surface potentials were calculated with PDB2PQR 3.6.2 server [48] at pH set to pH 7.5 value and apbs 3.4.1 suite [49] under default parameter values.

The electrostatic surface potential of SH2L_Ulv3 defined a positively charged patch in and around the active site pocket fitting with the negatively charged nature of the uronic acid substrate with sulphate substitutions (Fig. 9A,B). Compared to the PLSV_3936 ulvan lyase, the positive patch of SH2L_Ulv3 is less prominent, but the entry to the cleft on the β‐propeller surface of SH2L_Ulv3 is larger than that characterised in the PLSV_3936 ulvan lyase structure. The observed discrepancy is due to a loop located close to the K^+^ ion (Fig. 8) consisting of 11 residues N60‐PLAIVQHPA‐G70 in the PLSV_3936 structure, while 8 residues N36‐TITGNE‐G43 were modelled in the corresponding loop in the SH2L_Ulv3 structure model. The difference of three residues in the loop opens the middle of the protein β‐propeller fold creating a comparatively larger central cleft cavity in SH2L_Ulv3 compared to PLSV_3936 ulvan lyase (PDB 5UAM [14]).

Molecular docking simulations performing placement of two major ulvan disaccharide aldobiuronic acid moieties type A_3S_ (β‐d‐Glc*p*A‐(1 → 4)‐α‐l‐Rha3S) and type B_3S_ (α‐l‐IdoA‐(1 → 4)‐α‐l‐Rha3S) ulvanobiuronic‐3‐sulfates with the SH2L_Ulv3 structure model, respectively (Fig. S8A,B) and 5UAM chain B structure [14]. The orientation of the ulvan disaccharide poses (Table S1) outlined the relevance of the carboxylic group orientation related to the catalytic mechanism. For GlcA with a β‐d‐GlcA configuration, the C5 hydrogen and scissile bond oxygen point in the same direction, whereas for the α‐l‐IdoA, the C5 hydrogen points in the opposite direction of the oxygen in the scissile bond. The docking simulations of the ulvan disaccharide type B_3S_ revealed comparatively distant docking of the scissile bond and C5 hydrogen from the catalytic residues, whereas the scissile oxygen and the C5 proton for ulvan disaccharide type A_3S_ were predicted as docked in close proximity to the catalytic residues relevant for the β‐elimination (Fig. S8A,B).

## Discussion

The target SH2L_Ulv3 lyase was identified from the microbial metagenome enriched *in situ* on green and brown seaweed biomass in intertidal coastal hot springs located at the Icelandic coast as ulvan lyase active on a broad range of ulvans at a broad temperature range 15–55 °C with activity optimum at 20–25 °C demonstrating comparatively high polysaccharide degradation efficiency [17]. High heterologous (over)production yield of recombinant SH2L_Ulv3 remaining almost completely soluble in the expression host as well as after affinity purification outlined novel enzyme application potential as well.

Annotation of the short contig including the gene encoding SH2L_Ulv3 did not enable attribution to any particular species genome, but the SH2L_Ulv3 annotation confirmed homology to a sequence of a hypothetical protein from Bacteroidales bacterium identified from an aquatic metagenome [18]. Further extensive SH2L_Ulv3 sequence comparison did not result in any precise source identification, as SH2L_Ulv3 was homologous with identity not exceeding around 50% to various hypothetical BNR_4 proteins from phylogenetically somewhat different mostly marine bacteria strains. Comparison with metagenome sequencing data also revealed SH2L_Ulv3 sequence homology to a hypothetical protein from a marine hydrothermal vents sediment metagenome [20]. Phylogenetic grouping of SH2L_Ulv3 with homologous hypothetical BNR_4 protein sequences clustered SH2L_Ulv3 sequence with predominantly sequences from Flavobacteriaceae (order Flavobacteriales) as well as Flammeovirgaceae bacteria (order Cytophagales) from marine econiches, as well as the hypothetical Bacteroidales bacterium. Although the exact origin is not clear, it is apparent that the enzyme is distinctly different from the previously characterized enzymes in PL25 family.

The SH2L_Ulv3 initial attribution to PL25 family [17] could, however, be confirmed based on sequence and catalytic residues conservation, albeit the characterised lyase identity to characterised PL25 lyases was not exceeding sequence identities around 50%. Characterised PL25 ulvan lyases phylogenetic relation analysis grouped SH2L_Ulv3 as diverging from most identical PL25 lyases NLR_492 (IL45_03835) from *N. ulvanivorans* PLR [22] and ID172 from *A. luteus* P7‐3‐5 [16]. SH2L_Ulv3 sequence was notably more diverged comparing with characterised PL25 lyases. Comparison of SH2L_Ulv3 sequence with all nine characterised ulvan lyases comprising PL25 family [11] outlined enzyme novelty as well as distinct phylogenetic relationships.

Characterised PL25 lyases are secreted as single‐domain proteins consisting of a sole catalytic module. These features are fully reflected in the SH2L_Ulv3 sequence by confident prediction of the signal peptide and identification of the representative BNR repeat‐containing family member domain also defined with the synonymous Sialidase superfamily region as typical for PL25 family sequences. Analytical size exclusion chromatography confirmed monomer as the native characterised ulvan lyase state indirectly also indicating a compact structural organisation of the SH2L_Ulv3 monomer. The only PL25 lyase structure available was crystallised as two protein molecules in the asymmetric unit (PDB 5UAM and PDB 5UAS [14]). However, PLSV_3936 from *P*. sp. PLSV the only PL25 lyase crystallise is a monomer in its active form [14], as most typical for previously characterised PL25 family lyases [6, 25]. (Homo)oligomerisation was however also observed for purified NLR_492 (IL45_03835) from *N. ulvanivorans* PLR [22], but such oligomerisation or aggregation was not observed for SH2L_Ulv3 even at comparatively high pure protein concentration in between 2 and 4 mg·mL^−1^ in the analysis buffer. SH2L_Ulv3 also remained soluble even after prolonged incubations as well as freeze–thaw round after prolonged storage, indicating high solubility.

SH2L_Ulv3 activity characterisation using ulvan from *U. armoricana* (fine grade) as optimal [17] substrate characterised the activity of the extracellularly active secreted ulvan lyase as fully adapted to marine econiches rich in green algae biomass. SH2L_Ulv3 was most active over a narrow pH 7–8 range with optimum at pH 7.5 demonstrating activity over a broad alkaline pH range. The observed pH optimum also corresponds to the expected sea water pH 7.5–8 near the Icelandic coast [26, 27], while pH 7 was measured at the metagenome enrichment site during low tide [17]. The characterised ulvan lyases NLR_492 (IL45_03835) and ID172 that are most evolutionary related to SH2L_Ulv3 were also active at pH 7.5 [16, 22]. Characterised PL25 lyases are typically most active over a comparatively narrow pH range, albeit often covering a slightly more alkaline range pH 8–9, demonstrating an activity shift to an alkaline pH range [23, 25, 28]. Ulvan degrading activity of SH2L_Ulv3 was stable pH 5–9 and corroborates the typically high pH stability of PL25 lyases [6, 12, 29].

SH2L_Ulv3 was neither a thermoactive nor thermostable enzyme with an apparent temperature optimum at 25 °C remaining most active up to 30 °C and effectively active at 35–55 °C, while the lyase was not thermostable incubating for 30 min at temperatures exceeding 35–40 °C. Obvious adaptation to an expected temperature range suitable for seaweeds grown in the ocean was not surprising also taking into context the SH2L_Ulv3 origin.

The apparent temperature optimum of SH2L_Ulv3 is the lowest determined for a PL25 ulvan lyase as temperature optima of the most thermoactive PL25 lyases can reach 50–60 °C [22, 29]. However, the SH2L_Ulv3 thermoactivity corresponded to that of the ID172 ulvan lyase [16]. Moreover, thermostability was higher than the apparent thermoactivity, and PL25 lyases are typically demonstrating low thermostabilities not exceeding 1 h at 40–45 °C [6, 16, 28, 29]. Thermostability of PL25 lyases was previously only assessed by measurement of residual activity after preincubations, while thermostability of characterised ulvan lyase was also assessed by the continuous nanoDSF method which enabled definition of the unfolding temperature at 42 °C approximately 17 °C above the temperature optimum at 25 °C. Supposed as a result of thermoactivity and thermostability temperature comparison of PL25 ulvan lyases substrate stabilising effect by the ulvan polysaccharide [16, 22, 29] was fully confirmed by determination of *T*_m_ which at 0.5% (w/v) ulvan was increased to 47 °C, though stabilisation effect was not linear.

Metal ions are expected to be present in green algae biomass as well as in sea water [30] and their presence influences the SH2L_Ulv3 activity. The observed influence mostly corresponds to influences determined also for other PL25 ulvan lyases [6, 23, 25, 28] as Mg^2+^ ions at the lowest evaluated 1 mm concentration slightly stimulated activity, while Co^2+^, Ni^2+^, Cu^2+^, Zn^2+^ and Fe^2+^ or Fe^3+^ ions reduced enzyme activity to different extents. Ca^2+^ ion typically increasing activity of PL25 ulvan lyases had no significant effect on SH2L_Ulv3 activity. Minor but variable effects on PL25 enzymes have also been demonstrated for Mn^2+^ ions [6, 23, 25, 28] that had no substantial effect on SH2L_Ulv3. Ulvan degrading activity of SH2L_Ulv3 was reduced by Zn^2+^ ion at both 1 and 10 mm concentrations; however, 1 mm Zn^2+^ reduced activity to a lesser extent than 10 mm as could be expected though concentration‐dependent influence was nonlinear. The negative influence of Zn^2+^ may be surprising, as this metal ion is found in the crystal structure of the PL25 enzyme PLSV_3936 [14], however toxic effect of zinc for zinc‐dependent enzymes at higher than optimal ion concentration is expected [31]. Catalytic activity of PL25 ulvan lyases Zn^2+^ ion always reduces, albeit for SH2L_Ulv3 reducing influence was lower than for most other characterised PL25 ulvan lyases [22, 29]. Molar protein : zinc ratio 1 : 0.52, calculated based on zinc content in pure SH2L_Ulv3 sample measurement applying ICP‐OES, was not conclusive; however, it indirectly suggests Zn^2+^ ion coordination in the SH2L_Ulv3 monomer. Influence of divalent metal ion on SH2L_Ulv3 activity evaluation inconsistencies, as well as lack of chelation with EDTA influence on activity, could be caused as a result of quite substantial influence of metal ion adsorption capability and/or ions already adsorbed by ulvan polysaccharide [32]. Characterised SH2L_Ulv3 was efficiently active up to 500 mm NaCl demonstrating moderate salt tolerance with determined 200 mm as optimal NaCl concentration appearing typical for the environment of enzyme origin. Salt tolerance varies between characterised PL25 lyases from moderate tolerance at 150–200 mm to high tolerance up to 2 m of NaCl [6, 14, 22, 23, 28, 29].

Substrate specificity analysis confirmed SH2L_Ulv3's ability to disrupt ulvan polysaccharide from different green algae species of blade‐thallus morphology [17]. The characterised ulvan lyase also degraded ulvan from *E*. *intestinalis* of tubular‐thallus even though notably less efficiently than ulvan from *U. armoricana*, determined as optimal SH2L_Ulv3 substrate, on which the enzyme had a 10 times higher specific activity. A relatively broad substrate specificity is expected for PL25 ulvan lyases. Ulvan, as a high molecular weight complex branched polysaccharide, influences kinetic parameters of degradation reaction limiting direct comparison between different ulvan lyases. For the SH2L_Ulv3 reaction, *K*_M_ 3.63 ± 0.12 mg·mL^−1^ and *V*_max_ 1.78 ± 0.04 μmol·min^−1^·mL^−1^ were comparable with the parameters determined for the characterised PL25 lyases ULA‐2 and ULA‐3 from *Alteromonas* strains [6, 23], while SH2L_Ulv3 was less efficient with *k*_cat_ 1.46 ± 0.04 s^−1^, than TsUly25B lyase from *T*. sp. LD5, with a determined *k*_cat_ 10.52 ± 0.28 s^−1^ [29], which may be due to adaptations to their respective environments.

NMR identification of successfully separated ulvan degradation products confirmed SH2L_Ulv3 endo‐activity targeting polysaccharide backbone β‐(1 → 4)‐glycosidic linkages through a β‐elimination with the formation of an unsaturated bond at the nonreducing end of reaction products. DP2 identified as ∆‐(1 → 4)‐l‐Rhap3Sα/β was determined as the prevailing degradation product, along with three DP4 identified as ∆‐(1 → 4)‐α‐l‐Rhap3S‐(1 → 4)‐α‐l‐Ido*p*A‐(1 → 4)‐l‐Rhap3Sα/β, ∆‐(1 → 4)‐α‐l‐Rhap3S‐(1 → 4)‐β‐d‐Xyl*p*‐(1 → 4)‐l‐Rhap3Sα/β and ∆‐(1 → 4)‐α‐l‐Rha*p*3S‐(1 → 4)‐β‐d‐Glc*p*A‐(1 → 4)‐l‐Rha*p*3Sα/β that could be further cleaved into DP2. Confirmed activity manner, targeted linkages type as well as degradation products confirming a β‐elimination that would be expected for a PL25 ulvan lyase. Previously characterised PL25 lyases are reported to depolymerise ulvan polysaccharide into ulvan oligosaccharides of DP2 and DP4 with DP2 as the prevailing product of PL25 family activity [12].

Multiple sequence alignment of PL25 sequences revealed completely matching fully conserved active site residues, and several conserved binding site residues for SH2L_Ulv3 including zinc ion coordinating residues, what suggests zinc ion coordination and catalytic mechanism as well as substrate binding correspondence to characterised for PL25 family lyases [11, 14], despite the enzyme sequence more substantial phylogenetic distance. Predicted SH2L_Ulv3 catalytic site residues His95 and Tyr160, as expected, were confirmed by site‐directed mutagenesis as mutation of the latter amino acid residue to Ala completely inactivated the lyase. Observed aggregation of purified SH2L_Ulv3‐Tyr160Ala mutant also indirectly confirmed the importance of the fully conserved Tyr160 residue for enzyme structural organisation.

SH2L_Ulv3 AlphaFold3 structural model revealed the overall structural organisation of the enzyme as similar to the structure of PLSV_3936 from *P*. sp. PLSV [14] adopting β‐propeller fold similar to PLSV_3936 ulvan lyase as proposed for the PL25 family [6, 23, 25, 29]. SH2L_Ulv3 was 45% identical over 97% query coverage to PLSV_3936 lyase remaining the only PL25 lyase whose structure has been fully characterised. Structure superimposition revealed expected zinc ion coordination by residues His180, His240, Cys242, and His247 in SH2L_Ulv3 model, that displayed the expected catalytic residues His95 and Tyr160, and conserved substrate binding residues being completely congruent with the positions in the PLSV_3936 structure. The central active site region in the SH2L_Ulv3 was defined as an electrostatically positively charged patch enabling the fitting of negatively charged substrates. The electrostatic surface potential indicated that the active site cleft area in the predicted structural model of SH2L_Ulv3 was larger than characterised in the solved PLSV_3936 ulvan lyase structure. Molecular docking simulations confirmed the expected β‐elimination attack point illustrating the relevance of the carboxylic group orientation in both SH2L_Ulv3 and PLSV_3936 lyase.

SH2L_Ulv3 from an intertidal coastal hot spring algae biomass metagenome was confirmed as a functional ulvan lyase after cloning, production and purification. High production yield and stability of novel PL25 ulvan lyase with broad substrate specificity efficiently producing DP4 and DP2 products from ulvan polysaccharide prioritise implementation of SH2L_Ulv3 for oligosaccharide production from raw green algae biomass as well as for analytical applications. The ulvan lyase was active as a monomer at neutral to slightly alkaline pH at low temperatures up to 35 °C demonstrating salt tolerance up to 500 mm sodium chloride. The enzyme was stable at neutral to slightly alkaline pH and displayed an unfolding temperature *T*_m_ 42 °C. Most metal ions expected to be present in green algae biomass from sea water were not substantially reducing the activity of SH2L_Ulv3 lyase, while magnesium ion stimulated activity. The computationally modelled structure of SH2L_Ulv3 revealed structural organisation, active site architecture, confirmed by site‐directed mutagenesis, as well as ligand substrate binding residues were typical for PL25 lyases, however, with a larger central active site cleft facilitating ulvan polysaccharide degradation, making the enzyme an interesting candidate for ulvan oligosaccharide production. SH2L_Ulv3 implementation could be further developed by identifying long ulvan oligosaccharides produced at the initial phase of ulvan degradation, preference towards different DP4 substrates as well as the possibility to implement lyase along with other CAZymes achieving synergy of ulvan degradation.

## Materials and methods

### Enzyme sequence and phylogenetic analysis

SH2L_Ulv3 ulvan lyase encoded by *SH2L_Ulv3* gene domain organisation description [17] was refined with interproscan tool [33] based on Member Databases. Protein parameters were predicted with Expasy server protparam tool [34] and the signal peptides were predicted with SignalP 6.0 server [35]. Protein sequences after signal peptide omission were compared with blastp [36] implemented in ncbi blast [37] suite when necessary comparing SH2L_Ulv3 against nr, env_nr or pdb sequence databases as well as manually selected sequences under default parameter values. The mega11 suite [38] applying Jones–Taylor–Thornton matrix‐based model [39] was used to calculate maximum likelihood trees under default parameter values with 500 bootstrap replications [40]. ncbi blast suite output containing 65 most similar to SH2L_Ulv3 protein sequences accessible via nr database was extracted and re‐aligned after signal peptide omission and manual consideration with clustalw implemented in the mega11 suite under default parameter values for phylogenetic tree calculation. Multiple sequence alignments and phylogenetic tree analysing SH2L_Ulv3 phylogenetic relationships with characterised homologous ulvan lyases were generated analogously. The graphical representations of multiple sequence alignments with conserved residues as well as secondary structure elements indicated were created with ESPript 3.0 server [41] and the graphical representations of phylogenetic trees were created with itol v6 tool [42].

### Cloning, protein production and purification

Enzyme gene cloning was performed applying NEBuilder HiFi DNA Assembly (New England Biolabs). The selected in‐house expression vector pHWG1106 [17] based on rhamnose‐inducible vector pJOE4905.1 [43] was digested with *Nde*I and *Bam*HI, and subsequently, the linearised vector was purified from agarose gel using the Monarch DNA Gel Extraction Kit (New England Biolabs). The *SH2L_Ulv3* gene, omitting the signal peptide encoding sequence, was amplified by PCR with Q5 High‐Fidelity DNA Polymerase (New England Biolabs) from SH2L metagenome DNA using the gene‐specific primer pair SH2L_Ulv3_F: 5′‐CTTAAGAAGGAGATATACATATGCAGCCCAAACAGCTTG‐3′ and SH2L_Ulv3_R: 5′‐TTAATGATGATGATGATGATGGGATCCTTCATTTATTAGATCAGCTTCAACCTTAG‐3′ including overhangs compatible with the vector [17]. The amplicon from PCR was purified using the HighPrep PCR Clean‐up System (MagBio Genomics) and assembled C‐terminally incorporating a His_6_‐tag encoding sequence into the linearised vector performing standard NEBuilder HiFi DNA Assembly Master Mix (New England Biolabs) reaction. The assembly product was chemically transformed into *E. coli* NEB10‐beta (New England Biolabs). Transformants were cultivated overnight on LB‐Miller medium supplemented with 100 μg·mL^−1^ ampicillin subsequently inoculating culture for expression construct isolation from harvested cells using the Monarch Plasmid Miniprep Kit (New England Biolabs).

Site‐directed mutagenesis *SH2L_Ulv3‐His95Ala* and *SH2L_Ulv3‐Tyr160Ala* sequences were *de novo* synthesised C‐terminally incorporating a His_6_‐tag encoding sequence (Gene Universal). Sequence cloning was performed into pET‐21b(+) vector (Merck).

Enzyme gene expression construct pHG293 verified by sequencing (Fig. S9) was heat–shock transformed into *E. coli* JM109 (Promega). Expression culture inoculated with 1% (v/v) overnight culture was cultivated in LB‐Lennox medium supplemented with 100 μg·mL^−1^ ampicillin. Heterologous production of SH2L_Ulv3 was induced at OD_600_ 0.6–0.8 of expression culture with 0.15% (w/v) l‐rhamnose for 4 h at 30 °C. Site‐directed mutagenesis sequence expression constructs pET‐21b(+)::SH2L_Ulv3‐His95Ala and pET‐21b(+)::SH2L_Ulv3‐Tyr160Ala verified by sequencing (Fig. S9) were heat‐shock transformed into *E. coli* BL21(DE3) (Merck). Expression cultures inoculated with 1% (v/v) overnight culture were cultivated in LB‐Lennox medium supplemented with 100 μg·mL^−1^ ampicillin. Heterologous productions of SH2L_Ulv3‐His95Ala and SH2L_Ulv3‐Tyr160Ala mutants were induced at OD_600_ 0.6–0.8 of expression culture with 1 mm of IPTG for 4 h at 30 °C. Cells were subsequently harvested by centrifugation and lysed by ultra‐sonication using UP400S homogeniser (Hielscher Ultrasonics). The lysates were separated from cell debris by centrifugation at 26 000 ***g*** for 30 min at 4 °C. Obtained supernatants were filtered through regenerated cellulose 0.2 μm pore size filters (Cytiva).

SH2L_Ulv3 as well as SH2L_Ulv3‐His95Ala and SH2L_Ulv3‐Tyr160Ala were purified applying nickel affinity chromatography using HisTrap HP 1 mL (7 × 25 mm) columns (Cytiva) with ÄKTA start FPLC system (GE Healthcare Life Sciences). The His‐tagged proteins bound to resin in binding buffer (50 mm HEPES–NaOH pH 7.4/RT, 50 mm imidazole, 500 mm NaCl and 5% (v/v) glycerol) and were eluted (1–3 CV) after extensive washing with binding buffer (10 CV) with elution buffer (50 mm HEPES–NaOH pH 7.4/RT, 500 mm imidazole, 500 mm NaCl and 10% (v/v) glycerol). Buffer exchange to formulation buffer (50 mm HEPES–NaOH pH 7.4/RT and 10% (v/v) glycerol) was performed applying group separation using HiTrap Desalting 5 mL (16 × 25 mm) columns (Cytiva) loading 10% injection volume onto two tandem connected desalting columns at 0.5 mL·min^−1^ flowrate. Desalted proteins were filtered through regenerated cellulose 0.2 μm pore size filters (Cytiva). Obtained protein aliquots were supplemented with glycerol up to 20% (v/v) and flash‐frozen in liquid nitrogen. Recombinant protein integrity and purity were assessed by 4–15% glycine–SDS/PAGE. The protein concentration was determined considering recombinant protein ε_0.1%_ by measuring *A*_280_ using a BioSpec‐nano spectrophotometer (SHIMADZU).

### Analytical size exclusion chromatography

SH2L_Ulv3 molecular weight and potential oligomerisation were analysed applying analytical size exclusion chromatography using Superdex 200 Increase 10/300 GL column (Cytiva) with ÄKTA pure 25 L FPLC system (GE Healthcare Life Sciences). Ferritin (440 000 M_r_), aldolase (158 000 M_r_), conalbumin (75 000 M_r_), ovalbumin (43 000 M_r_), carbonic anhydrase (29 000 M_r_), ribonuclease A (13 700 M_r_) and aprotinin (6500 M_r_) protein standards (Cytiva) were used for column calibration determining void volume with Blue Dextran 2000 standard (Cytiva). Separation at 0.5 mL·min^−1^ was performed loading 0.4% injection volume and subsequently eluting with analysis buffer (50 mm HEPES–NaOH pH 7.4/RT and 25 mm NaCl). Prior to analysis, buffer exchange to analysis buffer was performed for samples applying group separation.

### Activity measurement

SH2L_Ulv3 ulvan degrading activity was assayed [17] spectrophotometrically by the dinitrosalicylic acid (DNS) assay [44] as well as by measuring *A*_235_ using Thermo Scientific Multiskan GO microplate reader (Thermo Fisher Scientific). The reaction of 250 μL final volume contained 0.5% (w/v) substrate and 1 μg enzyme in reaction buffer (50 mm HEPES–NaOH pH 7.5/RT). Activity was determined after incubation for 1 h at 25 °C. Formed free reducing ends of reaction products were measured as *A*_540_ after development at (v/v) ratio 1 : 1 with DNS reagent for 10 min at 100 °C. One unit (U) of ulvan lyase activity determined by the DNS assay was defined as the amount of enzyme required to produce 1 μmol of d‐xylose reducing sugar equivalent per min. Unsaturated ulvan products measured spectrophotometrically as *A*_235_ were quantified considering the extinction coefficient of the double bond (ε 4800 m ^−1^·cm^−1^). SH2L_Ulv3 inactivated by heating for 10 min at 100 °C served as a corresponding control. One unit (U) of ulvan lyase activity determined by measuring unsaturated products was defined as the amount of enzyme required to produce 1 μmol of unsaturated products per min. All measurements were performed in triplicates.

### Thin‐layer chromatography

The reactions were fractionated with TLC and stained to visualise the reaction products. Two microlitre of reaction were air‐dry loaded on a TLC Silica gel 60 F_254_ plate (Merck) subsequently resolving with a mobile phase composed of n‐butanol, acetic acid, and water at (v/v/v) ratio 2 : 1 : 1, respectively. l‐rhamnose as well as d‐xylose were used as migration reference standards. The developed plates were air‐dried and stained by spraying with staining solution (5% (v/v) sulfuric acid in methanol supplemented with 1 mg·mL^−1^ orcinol) followed by heating on a hotplate at 110 °C.

### Activity optima and substrate specificity determination

SH2L_Ulv3 activity optimum was evaluated with 0.5% (w/v) ulvan from *U*. *armoricana* (fine grade) (ULV102; ELICITYL) used as substrate. The pH optimum of SH2L_Ulv3 activity was determined over a pH range of pH 5–9 in buffers (50 mm sodium acetate–acetic acid pH 5/RT, 50 mm MES–NaOH pH 6/RT, 50 mm HEPES–NaOH pH 7/RT and pH 7.5/RT, 50 mm Tris/HCl pH 8/RT as well as 50 mm glycine–NaOH pH 9/RT) measuring ulvan degrading activity at 30 °C. The temperature optimum of SH2L_Ulv3 activity was determined over a temperature range of 15–65 °C in reaction buffer measuring ulvan degrading activity. The pH stability of SH2L_Ulv3 was determined measuring residual activity in reaction buffer at optimal temperature after enzyme aliquots preincubation in buffers of pH 5–9 for 30 min. SH2L_Ulv3 thermostability was determined by measuring residual activity in reaction buffer at optimal temperature after enzyme aliquots preincubation at 15–65 °C in reaction buffer for 30 min and at 95 °C for 10 min. Cooling for 10 min on ice was performed after aliquot preincubation for residual activity measurements.

Influence of divalent metal ion on SH2L_Ulv3 activity was evaluated by performing optimal ulvan degrading activity reaction in reaction buffer supplemented with 1 and 10 mm MgCl_2_, CaCl_2_, MnCl_2_, FeCl_2_, CoCl_2_, NiCl_2_, CuCl_2_ or ZnCl_2_ after reaction mixture preincubation, before adding substrate, at optimal temperature for 30 min. The ulvan degrading activity measured supplementing enzyme optimal activity reaction with 1 or 10 mm EDTA served as a corresponding control. Influence of salt, including 50, 100, 200, 500 and 2000 mm NaCl in reactions, determining SH2L_Ulv3 activity was also evaluated.

SH2L_Ulv3 substrate specificity was evaluated by performing optimal ulvan degrading activity reaction with 0.5% (w/v) ulvan from *U*. *armoricana* (fine grade) or 0.5% (w/v) *E*. *intestinalis* (fine grade) (ULV103; ELICITYL). Optimal activity reaction evaluating substrate specificity was also performed at up to 3, 24 and 48 h prolonged incubations.

The ulvan degrading activity was measured by the DNS assay as already described. All measurements were performed in triplicates.

### Thermostability determination

SH2L_Ulv3 thermostability was estimated in formulation buffer applying nanoDSF using a Prometheus NT.48 instrument (NanoTemper Technologies). Standard grade glass capillaries (NanoTemper Technologies) were filled with enzyme solution at a concentration of 0.1 or 0.2 mg·mL^−1^ without substrate as well as at a concentration of 0.1 mg·mL^−1^ supplemented with 0.05 or 0.5% (w/v) ulvan from *U*. *armoricana* (fine grade). Thermal unfolding was performed at 40% excitation power with a temperature gradient between 15 °C and 95 °C at a 1 °C·min^−1^ ramp rate. *T*_m_ was determined from the first derivative of the absorbance ratio 350/330 nm and was interpreted automatically by the instrument pr.thermcontrol v2.0.4 software (NanoTemper Technologies). All measurements were performed in triplicates.

### Enzyme kinetics

SH2L_Ulv3 kinetic parameters were determined by performing optimal activity reaction with 0.01, 0.05, 0.1, 0.25, 0.5, 0.75 and 1% (w/v) ulvan from *U*. *armoricana* (fine grade) used as substrate in optimal reaction buffer (50 mm HEPES–NaOH pH 7.5/RT and 200 mm NaCl). Activity was assayed spectrophotometrically as *A*_235_ in triplicates. The *K*_M_ and *V*_max_ parameters were calculated by fitting measured data to the Michaelis–Menten equation applying a sum of least squares nonlinear regression [45] with Microsoft Excel implementing Solver add‐in program (Microsoft). The *k*_cat_ was subsequently also calculated.

### Inductively coupled plasma optical emission spectroscopy

Zinc content in pure SH2L_Ulv3 sample was quantified applying ICP‐OES using Optima 8300 Optical Emission Spectrometer (PerkinElmer). Double desalted recombinant protein was lyophilized by freeze drying (LABCONCO) and subsequently digested with nitric acid ensuring full decomposition by heating [46]. Digested SH2L_Ulv3 sample was diluted to 10% (v/v) nitric acid concentration with ultrapure water (Milli‐Q grade). Measurements were performed under ICP‐OES standard conditions detecting zinc at 206.200 nm in protein sample as well as corresponding controls. Zinc concentration was estimated by comparing with calibration and eventually calculated to molar protein : zinc ratio. All measurements were performed in triplicates.

### Reaction product purification

SH2L_Ulv3 reaction products from ulvan degradation were purified applying preparative size exclusion chromatography. Oligosaccharide fraction was purified from SH2L_Ulv3 optimal reaction performed at up to 24 h prolonged incubation using Superdex 30 Prep Grade resin (Cytiva) in XK 16/100 column (GE Healthcare Life Sciences) with Agilent 1100 Series HPLC system (Agilent Technologies). Dextran 5000 analytical standard (Merck) and d‐glucose with NaCl were used for packed column calibration determining void volume with Dextran 12 000 analytical standard (Merck). Purification at 0.3 mL·min^−1^ was performed loading 2% injection volume and subsequently eluting with ultrapure water (Milli‐Q grade). Fractions containing oligosaccharides after TLC visualisation were combined and lyophilized by freeze drying. Oligosaccharide fractions were separated using Bio‐Gel P‐2 resin (Bio‐Rad) in Econo‐Column (15 × 1000 mm) (Bio‐Rad) eluting with 50 mm ammonium carbonate. Fractions containing separated individual oligosaccharides after TLC visualisation were lyophilized by freeze drying for identification applying NMR spectroscopy.

### Nuclear magnetic resonance spectroscopy

Resolution‐enhanced 1D/2D 600 MHz ^1^H NMR spectra and 150 MHz ^13^C NMR spectra were recorded in D_2_O using an Avance Neo spectrometer (Bruker) equipped with a TCI Prodigy CryoProbe (Bruker) (Utrecht University, the Netherlands) at a probe temperature of 298 K. Prior to analysis, samples were exchanged twice in D_2_O (99.9 atom % D) (Cambridge Isotope Laboratories) with intermediate lyophilisation and then dissolved in 0.5 mL D_2_O. Chemical shifts (δ) were expressed in p.p.m. by reference to internal acetone (δ 2.225 (^1^H) and δ 31.07 (^13^C)). Suppression of HOD signal was achieved by applying a water‐eliminated Fourier transform (WEFT) pulse sequence for 1D NMR experiments and by a presaturation of 1 s during the relaxation delay in 2D NMR experiments. 2D TOCSY, NOESY, HMBC and HSQC spectra were acquired using standard pulse sequences (Bruker).

### Enzyme structure modelling and ligand docking simulations

Structural modelling of SH2L_Ulv3 was conducted with the AlphaFold3 model [47] for enzyme sequence query Gln20‐Glu453 after signal peptide omission as well as, additionally, including a single Zn^2+^ ion in query. The graphical visualisations of the structure models were created with the pymol 3.1 system (Schrödinger). The electrostatic surface potentials were calculated with PDB2PQR 3.6.2 server [48] at pH set to pH 7.5 value and apbs 3.4.1 suite [49] under default parameter values. Modelled structures were aligned with PLSV_3936 ulvan lyase PDB 5UAM and PDB 5UAS [14] structures superimposing with the pymol system.

Molecular docking simulations were conducted with the autodock vina 1.2.5 program [50, 51] preparing receptors and ligands with mgltools 1.5.7 suite AutoDock Tools [50]. The ligands pdbqt files were prepared by adding hydrogen atoms and charges. The SH2L_Ulv3 pdbqt receptor file was prepared by adding hydrogen atoms and charges, while PDB 5UAM chain B receptor pdbqt file was prepared by removing water molecules, phosphate ion, glycerol, 1,2‐ethanediol, K^+^ and Cl^−^ ions before adding hydrogen atoms and charges. Docking was performed with 10 poses, energy difference of 3 and exhaustiveness set to 32. The grid was manually placed in the active site location of the models with a grid size of *x* = 40 Å, *y* = 40 Å and *z* = 40 Å.

## Conflict of interest

The authors declare no conflict of interest.

## Author contributions

AJ and ENK designed the study. PS and AJ performed biophysical and biochemical characterisation. JMD performed NMR identification. SF performed bioinformatic analysis, supervised by ASM. HG and BTA performed gene cloning and initial activity screening, supervised by GÓH. AJ prepared the manuscript with input from all authors, supervised by ENK.

## Supporting information


**Fig. S1.** SH2L_Ulv3 molecular weight as well as oligomerization analysis applying analytical size exclusion chromatography including recombinant protein assessment by SDS–PAGE.
**Fig. S2.** SH2L_Ulv3 reaction of ulvan from *Ulva armoricana* (fine grade) degradation product purification applying size‐exclusion chromatography.
**Fig. S3.** 600 MHz 1D ^1^H NMR and 2D ^13^C–^1^H HSQC spectra of fraction I sample recorded in D_2_O at 298 K.
**Fig. S4.** 600 MHz 1D ^1^H NMR and 2D ^13^C–^1^H HSQC spectra of fraction II sample recorded in D_2_O at 298 K.
**Fig. S5.** 600 MHz 1D ^1^H NMR and 2D ^13^C–^1^H HSQC spectra of fraction III sample recorded in D_2_O at 298 K.
**Fig. S6.** 600 MHz 1D ^1^H NMR and 2D ^13^C–^1^H HSQC spectra of fraction IV sample recorded in D_2_O at 298 K.
**Fig. S7.** SH2L_Ulv3 ulvan lyase structure AlphaFold3 model structural alignment with PLSV_3936 ulvan lyase from *Pseudoalteromonas* sp. PLSV superimposing with PDB 5UAM chain B presented in cyan and PDB 5UAS chain B presented in grey structures.
**Fig. S8.** Molecular docking simulations of type A_3S_ (β‐d‐Glc*p*A‐(1 → 4)‐α‐l‐Rha3S) and type B_3S_ (α‐l‐IdoA‐(1 → 4)‐α‐l‐Rha3S) ulvan disaccharide aldobiuronic acid moieties ulvanobiuronic‐3‐sulfates with SH2L_Ulv3 structure model representing affinity pose −7.9 and −8.4 kcal·mol^−1^, respectively.
**Fig. S9.** Recombinant SH2L_Ulv3 sequence produced by using protein expression construct pHG293.1 as well as sequences of SH2L_Ulv3 mutants produced by using constructs pET‐21b(+)::SH2L_Ulv3‐His95Ala and pET‐21b(+)::SH2L_Ulv3‐Tyr160Ala.
**Table S1.** Top molecular docking poses of SH2L_Ulv3 structure model and 5UAM chain B with two ulvan disaccharide aldobiuronic acid moieties type A_3S_ (β‐d‐Glc*p*A‐(1 → 4)‐α‐l‐Rha3S) and type B_3S_ (α‐l‐IdoA‐(1 → 4)‐α‐l‐Rha3S) ulvanobiuronic‐3‐sulfates.

## Data Availability

All data supporting the findings of this study are included within the article. The generated protein structure models can be obtained by making a request to the corresponding author.
