# Supplementary material for: Characterisation of a phylogenetically distinct PL25 family ulvan lyase from a seaweed biomass enriched metagenome
Source: FEBS J. 2026 Jan 21;293(10):2885–907. doi: 10.1111/febs.70390 (PMC13193363; doi:10.1111/febs.70390)
Supplement: Supplementary file 1 — Fig. S1. SH2L_Ulv3 molecular weight as well as oligomerization analysis applying analytical size exclusion chromatography including recombinant protein assessment by SDS–PAGE. Fig. S2. SH2L_Ulv3 reaction of ulvan from Ulva armoricana (fine grade) degradation product purification applying size‐exclusion chromatography. Fig. S3. 600 MHz 1D 1H NMR and 2D 13C–1H HSQC spectra of fraction I sample recorded in D2O at 298 K. Fig. S4. 600 MHz 1D 1H NMR and 2D 13C–1H HSQC spectra of fraction II sample recorded in D2O at 298 K. Fig. S5. 600 MHz 1D 1H NMR and 2D 13C–1H HSQC spectra of fraction III sample recorded in D2O at 298 K. Fig. S6. 600 MHz 1D 1H NMR and 2D 13C–1H HSQC spectra of fraction IV sample recorded in D2O at 298 K. Fig. S7. SH2L_Ulv3 ulvan lyase structure AlphaFold3 model structural alignment with PLSV_3936 ulvan lyase from Pseudoalteromonas sp. PLSV superimposing with PDB 5UAM chain B presented in cyan and PDB 5UAS chain B presented in grey structures. Fig. S8. Molecular docking simulations of type A3S (β‐d‐GlcpA‐(1 → 4)‐α‐l‐Rha3S) and type B3S (α‐l‐IdoA‐(1 → 4)‐α‐l‐Rha3S) ulvan disaccharide aldobiuronic acid moieties ulvanobiuronic‐3‐sulfates with SH2L_Ulv3 structure model representing affinity pose −7.9 and −8.4 kcal·mol−1, respectively. Fig. S9. Recombinant SH2L_Ulv3 sequence produced by using protein expression construct pHG293.1 as well as sequences of SH2L_Ulv3 mutants produced by using constructs pET‐21b(+)::SH2L_Ulv3‐His95Ala and pET‐21b(+)::SH2L_Ulv3‐Tyr160Ala. Table S1. Top molecular docking poses of SH2L_Ulv3 structure model and 5UAM chain B with two ulvan disaccharide aldobiuronic acid moieties type A3S (β‐d‐GlcpA‐(1 → 4)‐α‐l‐Rha3S) and type B3S (α‐l‐IdoA‐(1 → 4)‐α‐l‐Rha3S) ulvanobiuronic‐3‐sulfates. [file FEBS-293-2885-s001.pdf]

SUPPORTING INFORMATION

Supplementary Figure 1. SH2L_Ulv3 molecular weight as well as oligomerization analysis applying analytical size exclusion chromatography including recombinant protein assessment by SDS–PAGE. Ferritin (440000 M_r), aldolase (158000 M_r), conalbumin (75000 M_r), ovalbumin (43000 M_r) carbonic anhydrase (29000 M_r), ribonuclease A (13700 M_r), and aprotinin (6500 M_r) protein standards (Cytiva) were used for column calibration determining void volume with Blue Dextran 2000 standard (Cytiva). Protein elution volume is indicated in red and column void volume is indicated in black. MW, molecular weight.

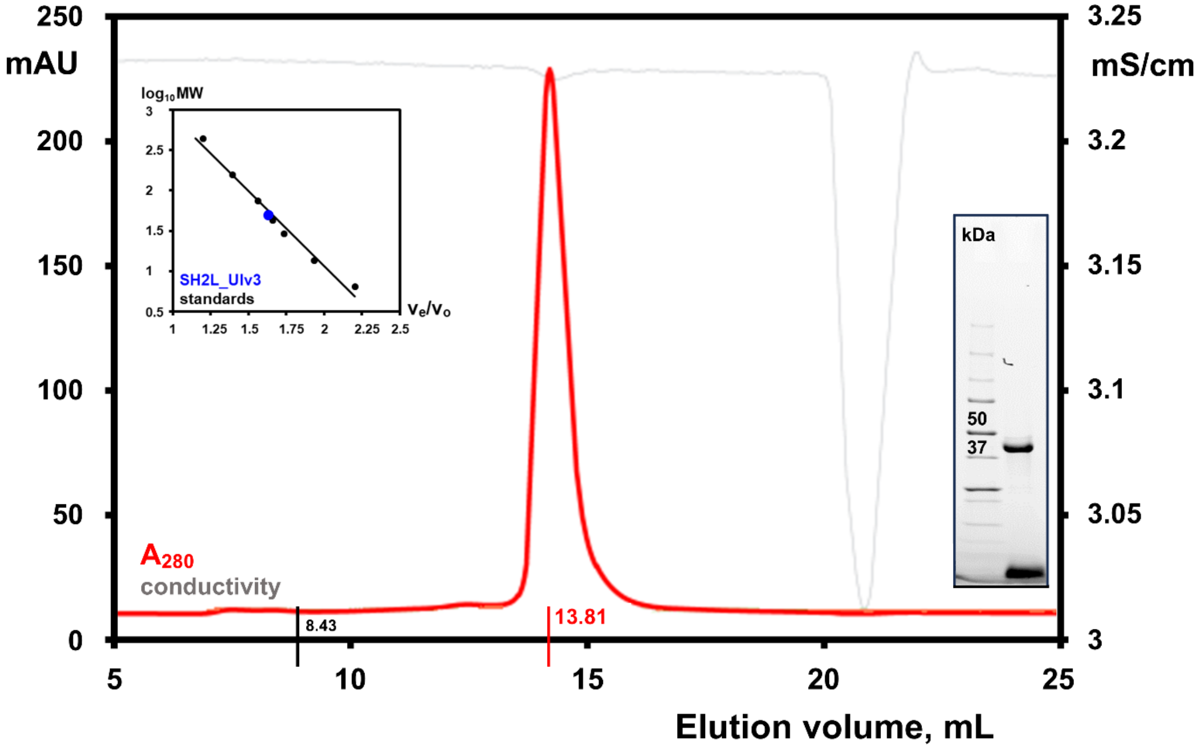

Supplementary Figure 2. SH2L_Ulv3 reaction of ulvan from *Ulva armoricana* (fine grade) degradation product purification applying size-exclusion chromatography. (**A**) Optimal activity reaction performed at prolonged up to 24 h incubation (U-F) ulvan as well as (PDU-F) partially degraded ulvan and (O-F)

oligosaccharide fraction separation; (**B**) Fraction TLC visualization. (Ua-R) Optimal activity reaction with ulvan from *U. armoricana* fractionation, (Rha) L-rhamnose, (Xyl) D-xylose, (C) Ulvan from *U. armoricana*. Packed column calibration was performed using Dextran 5000 analytical standard (Merck) and D-glucose with NaCl determining void volume with Dextran 12000 analytical standard (Merck). DP, degree of polymerisation.

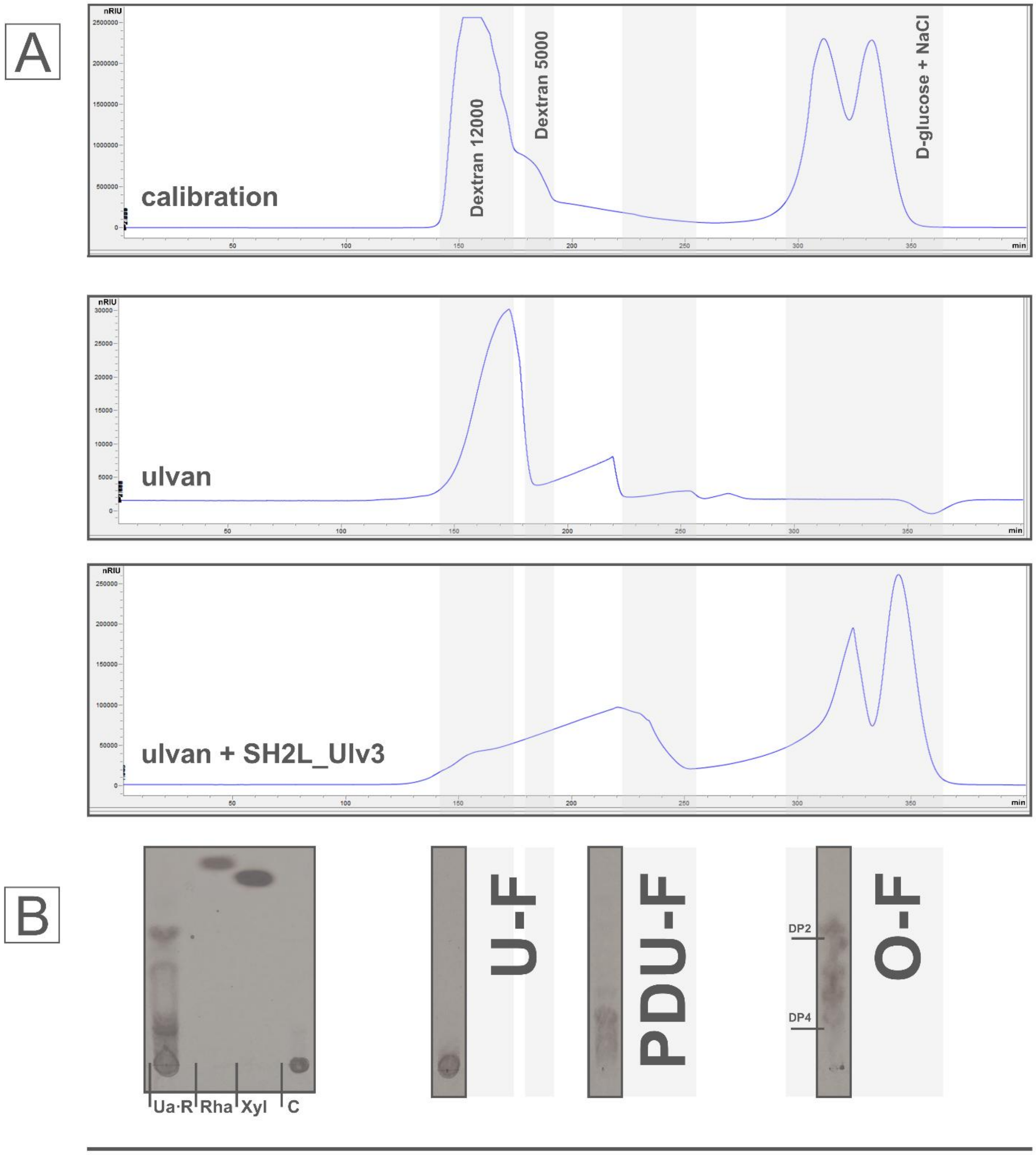

Supplementary Figure 3. 600 MHz 1D ^{1}H NMR and 2D ^{13}C–^{1}H HSQC spectra of fraction I sample recorded in D_2O at 298 K.

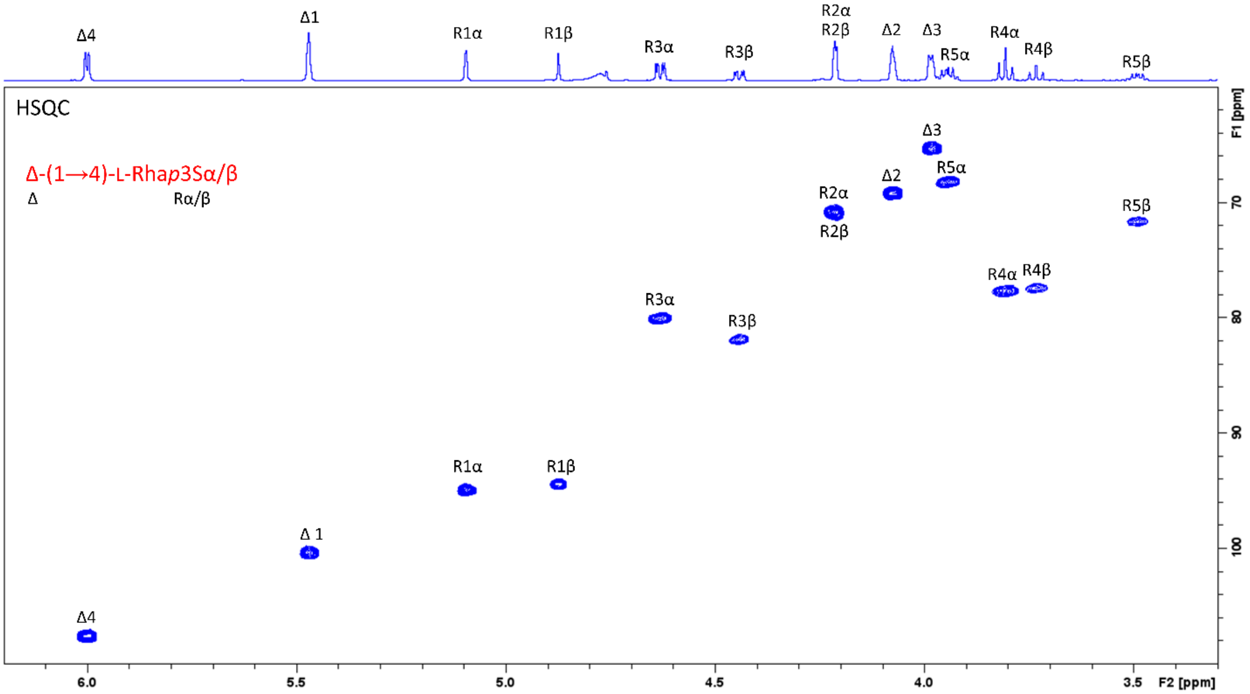

Supplementary Figure 4. 600 MHz 1D ^{1}H NMR and 2D ^{13}C–^{1}H HSQC spectra of fraction II sample recorded in D_2O at 298 K.

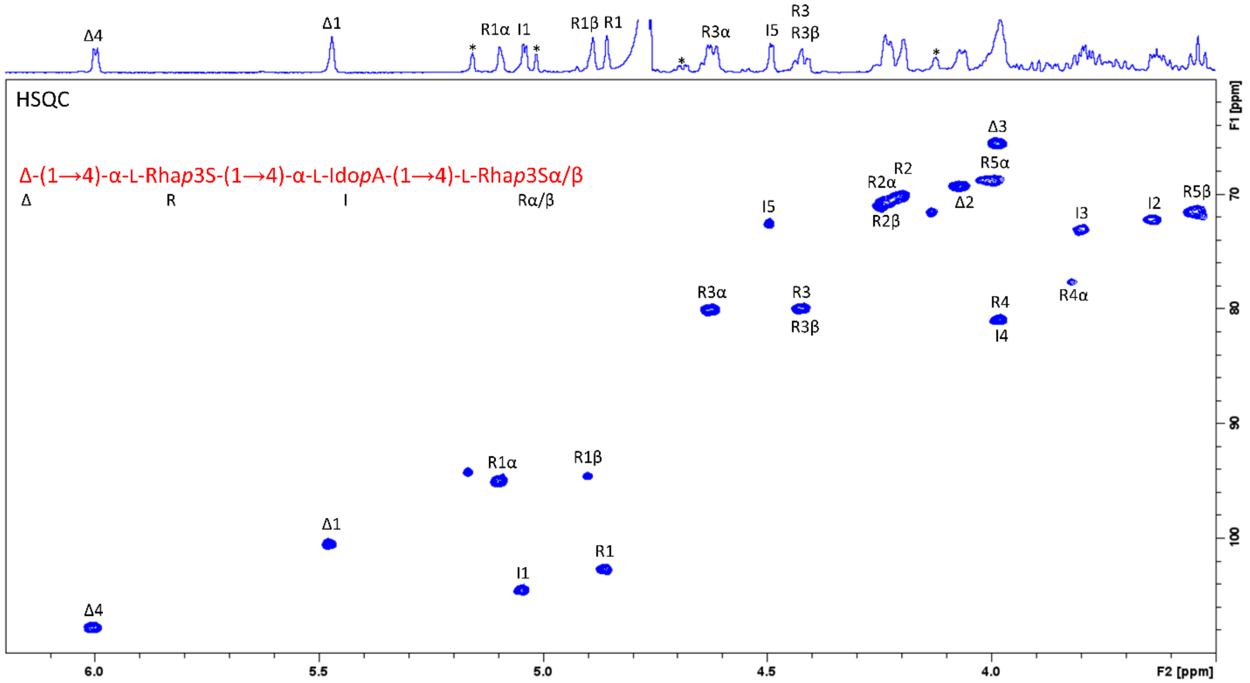

*minor quantities of other oligosaccharides.

Supplementary Figure 5. 600 MHz 1D ^{1}H NMR and 2D ^{13}C–^{1}H HSQC spectra of fraction III sample recorded in D_2O at 298 K.

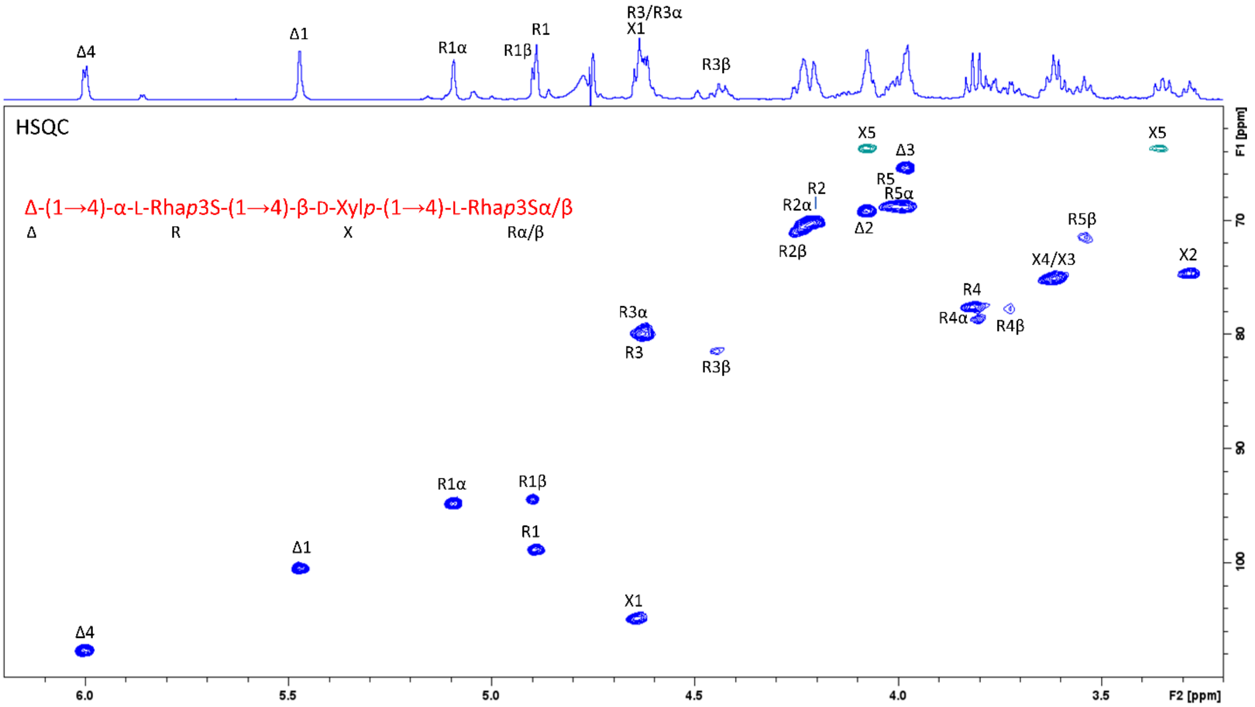

Supplementary Figure 6. 600 MHz 1D ^{1}H NMR and 2D ^{13}C–^{1}H HSQC spectra of fraction IV sample recorded in D_2O at 298 K.

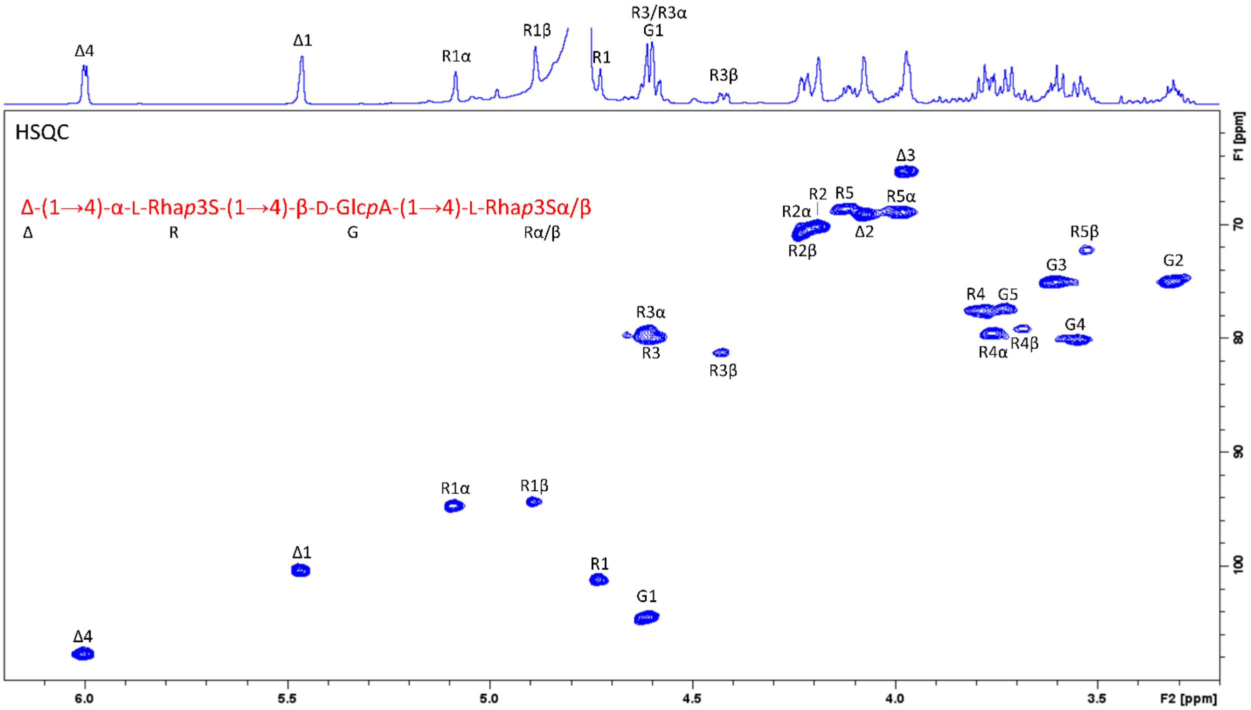

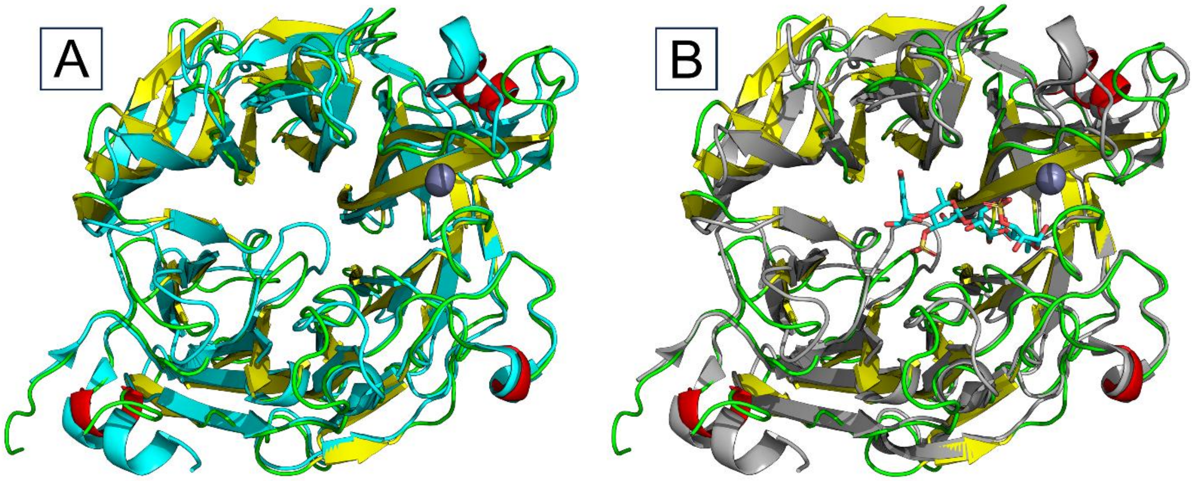

Supplementary Figure 7. SH2L_Ulv3 ulvan lyase structure AlphaFold3 model structural alignment with PLSV_3936 ulvan lyase from *Pseudoalteromonas* sp. PLSV superimposing with (**A**) PDB 5UAM chain B presented in cyan and (**B**) PDB 5UAS chain B presented in grey structures [14]. Sheets, helices and loops in SH2L_Ulv3 structure model are presented in yellow, red and green, respectively, while Zn^{2+} is presented in dark grey. PDB 5UAS chain B structure includes Δ-(1→4)-α-L-Rha*p*3S-(1→4)-β-D-Glc*p*A-(1→4)-α-L-Rha*p*3S as a ligand coloured by element with carbon in cyan. Structural modelling was conducted with the AlphaFold3 model. Graphical visualizations of the structure models, as well as structure superimpositions, were created with the PyMOL 3.1 system (Schrödinger).

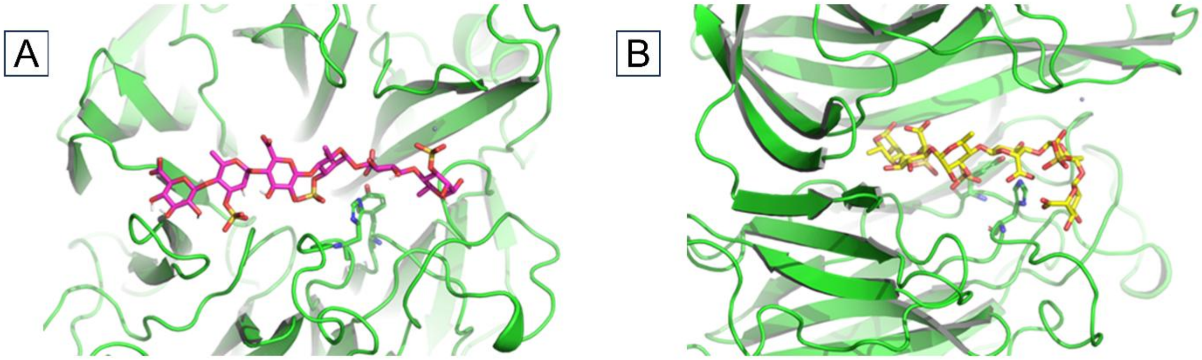

Supplementary Figure 8. Molecular docking simulations of (**A**) type A_{3S} (β-D-Glc*p*A-(1→4)-α-L-Rha3S) and (**B**) type B_{3S} (α-L-IdoA-(1→4)-α-L-Rha3S) ulvan disaccharide aldobiuronic acid moieties ulvanobiuronic-3-sulfates with SH2L_Ulv3 structure model representing affinity pose -7.9 kcal/mol and -8.4 kcal/mol, respectively. Structural modelling was conducted with the AlphaFold3 model. Molecular docking simulations were conducted with the AutoDock-Vina 1.2.5 preparing receptors and ligands with MGLTools 1.5.7 suite AutoDock Tools. Graphical visualizations of the ligand docking were created with the PyMOL 3.1 system (Schrödinger).

Supplementary Figure 9. Recombinant SH2L_Ulv3 sequence produced by using protein expression construct pHG293.1 as well as sequences of SH2L_Ulv3 mutants produced by using constructs pET-21b(+)::SH2L_Ulv3-His95Ala and pET-21b(+)::SH2L_Ulv3-Tyr160Ala. His_6-tag sequence outlined in yellow. Mutated amino acids residues outlined in red.

>SH2L_Ulv3 recombinant variant, pHG293.1
MQPKQLEVDYFANNGFGNTITGNEGEYYKGITYVCYQGPMEDPYVAAYDHKHKKWIGPYKAGTSLLGKTQN
KKIDNHGKPTLVVDGEGYIHIVFGGHGGTKDLGENTLGNYHAGKQMHVVSKEPMDISSWEVVDNISPFGTY
SQFLKMDNGDIYLFYRHGAHRSNWVYQVSKDNCRTFSPLVSIVKTKQIEGTVENPVIMDSWYLSFTLGEGN
DVIVAYNYHVCKGPHHDGERHNCYYMKFDTDKEQWLNVKGEMLELPITKEYADTMTLVKNTGDNWTHNGTA
MLDAQGMPHVSSYEGADDNTVHGGRKTIQHYYWDGTAWTATNTGLPEGAKGLMQAESAQNVNFLLGYTQGD
LAAIAWWKSVDGAKSFECGEHILSEKDANYSLTNMIRNAHPDARVIAAKKISGTDFSELYVIGDNGPIKRA
KVEADLINEGSHHHHHHH*

>SH2L_Ulv3-H95A mutant variant, pET-21b(+)::SH2L_Ulv3-H95A
MQPKQLEVDYFANNGFGNTITGNEGEYYKGITYVCYQGPMEDPYVAAYDHKHKKWIGPYKAGTSLLGKTQN
KKIDNAGKPTLVVDGEGYIHIVFGGHGGTKDLGENTLGNYHAGKQMHVVSKEPMDISSWEVVDNISPFGTY
SQFLKMDNGDIYLFYRHGAHRSNWVYQVSKDNCRTFSPLVSIVKTKQIEGTVENPVIMDSWYLSFTLGEGN
DVIVAYNYHVCKGPHHDGERHNCYYMKFDTDKEQWLNVKGEMLELPITKEYADTMTLVKNTGDNWTHNGTA
MLDAQGMPHVSSYEGADDNTVHGGRKTIQHYYWDGTAWTATNTGLPEGAKGLMQAESAQNVNFLLGYTQGD
LAAIAWWKSVDGAKSFECGEHILSEKDANYSLTNMIRNAHPDARVIAAKKISGTDFSELYVIGDNGPIKRA
KVEADLINEGSHHHHHHH*

>SH2L_Ulv3-Y160A mutant variant, pET-21b(+)::SH2L_Ulv3-Y160A
MQPKQLEVDYFANNGFGNTITGNEGEYYKGITYVCYQGPMEDPYVAAYDHKHKKWIGPYKAGTSLLGKTQN
KKIDNHGKPTLVVDGEGYIHIVFGGHGGTKDLGENTLGNYHAGKQMHVVSKEPMDISSWEVVDNISPFGTA
SQFLKMDNGDIYLFYRHGAHRSNWVYQVSKDNCRTFSPLVSIVKTKQIEGTVENPVIMDSWYLSFTLGEGN
DVIVAYNYHVCKGPHHDGERHNCYYMKFDTDKEQWLNVKGEMLELPITKEYADTMTLVKNTGDNWTHNGTA
MLDAQGMPHVSSYEGADDNTVHGGRKTIQHYYWDGTAWTATNTGLPEGAKGLMQAESAQNVNFLLGYTQGD
LAAIAWWKSVDGAKSFECGEHILSEKDANYSLTNMIRNAHPDARVIAAKKISGTDFSELYVIGDNGPIKRA
KVEADLINEGSHHHHHHH*

Supplementary Table 1. Top molecular docking poses of SH2L_Ulv3 structure model and 5UAM chain B with two ulvan disaccharide aldobiuronic acid moieties type A_{3S} (β-D-Glc*p*A-(1→4)-α-L-Rha3S) and type B_{3S} (α-L-IdoA-(1→4)-α-L-Rha3S) ulvanobiuronic-3-sulfates.

Pose	Affinity, kcal·mol^{-1}			
	SH2L_Ulv3 model		5UAM chain B [14]	
	Ulvan disaccharide			
	type A_{3S}	type B_{3S}	type A_{3S}	type B_{3S}
1	-8.1	-8.4	-8.5	-8.1
2	-8.0	-8.4	-8.4	-8.0
3	-8.0	-8.4	-8.3	-8.0
4	-7.9	-8.4	-8.3	-8.0
5	-7.9	-8.4	-8.2	-7.9
6	-7.9	-8.4	-8.2	-7.9
7	-7.8	-8.4	-8.1	-7.9
8	-7.8	-8.4	-8.1	-7.9
9	-7.7	-8.3	-8.0	-7.8
10	-7.7	-8.3	-8.0	-7.8
